# Value of Routine Dengue Diagnostic Tests in Urine and Saliva Specimens

**DOI:** 10.1371/journal.pntd.0004100

**Published:** 2015-09-25

**Authors:** Anne-Claire Andries, Veasna Duong, Sowath Ly, Julien Cappelle, Kim Srorn Kim, Patrich Lorn Try, Sopheaktra Ros, Sivuth Ong, Rekol Huy, Paul Horwood, Marie Flamand, Anavaj Sakuntabhai, Arnaud Tarantola, Philippe Buchy

**Affiliations:** 1 Institut Pasteur in Cambodia, Virology Unit, Phnom Penh, Cambodia; 2 Institut Pasteur in Cambodia, Epidemiology and Public Health Unit, Phnom Penh, Cambodia; 3 Centre de Coopération Internationale en Recherche Agronomique pour le Développement (CIRAD), Unité AGIRs, Montpellier, France; 4 Kampong Cham Provincial Hospital, Pediatric Department, Kampong Cham, Cambodia; 5 Ministry of Health, Centre National de Malariologie, Phnom Penh, Cambodia; 6 Institut Pasteur, Structural Virology Unit & CNRS UMR 3569, Paris, France; 7 Institut Pasteur, Functional Genetics of Infectious Diseases Unit, Paris, France; 8 Centre National de la Recherche Scientifique, Unité de Recherche Associée 3012, Paris, France; 9 GlaxoSmithKline Vaccines, Vaccine Value and Health Sciences, Singapore, Singapore; University of Pittsburgh, UNITED STATES

## Abstract

**Background:**

Dengue laboratory diagnosis is essentially based on detection of the virus, its components or antibodies directed against the virus in blood samples. Blood, however, may be difficult to draw in some patients, especially in children, and sampling during outbreak investigations or epidemiological studies may face logistical challenges or limited compliance to invasive procedures from subjects. The aim of this study was to assess the possibility of using saliva and urine samples instead of blood for dengue diagnosis.

**Methodology/Principal Findings:**

Serial plasma, urine and saliva samples were collected at several time-points between the day of admission to hospital until three months after the onset of fever in children with confirmed dengue disease. Quantitative RT-PCR, NS1 antigen capture and ELISA serology for anti-DENV antibody (IgG, IgM and IgA) detection were performed in parallel on the three body fluids. RT-PCR and NS1 tests demonstrated an overall sensitivity of 85.4%/63.4%, 41.6%/14.5% and 39%/28.3%, in plasma, urine and saliva specimens, respectively. When urine and saliva samples were collected at the same time-points and tested concurrently, the diagnostic sensitivity of RNA and NS1 detection assays was 69.1% and 34.4%, respectively. IgG/IgA detection assays had an overall sensitivity of 54.4%/37.4%, 38.5%/26.8% and 52.9%/28.6% in plasma, urine and saliva specimens, respectively. IgM were detected in 38.1% and 36% of the plasma and saliva samples but never in urine.

**Conclusions:**

Although the performances of the different diagnostic methods were not as good in saliva and urine as in plasma specimens, the results obtained by qRT-PCR and by anti-DENV antibody ELISA could well justify the use of these two body fluids to detect dengue infection in situations when the collection of blood specimens is not possible.

## Author Summary

Dengue is the most important arthropod-borne disease affecting humans and represents a huge public health burden in affected countries. Symptoms are often non-specific hence the need for an early, sensitive and specific diagnosis of dengue for appropriate management as well as for early epidemic detection. Currently, almost all laboratory diagnostic methods require a blood specimen that may be sometimes be difficult or inconvenient to obtain. In this study, we assessed the possibility to use saliva and urine samples as alternatives to blood specimens in dengue diagnosis. We demonstrated that the performances of the different diagnostic methods (RT-PCR, NS1 antigen detection and anti-DENV IgM/IgG/IgA ELISAs) were in general not as good in saliva and urine as in plasma, but that the use of these body fluids obtained by non-invasive methods could be of value in certain circumstances such as outbreak investigations or in young children (once they are old enough to comply to instructions), in addition to the situations when blood cannot be easily collected (e.g., lack of phlebotomist, refusal of the procedure, etc.).

## Introduction

Dengue virus (DENV; family *Flaviviridae*, genus *Flavivirus*), is a mosquito-borne, enveloped, single stranded positive-sense RNA virus. Until recently, four serologically related but antigenically and genetically distinct dengue viruses (DENV-1, -2, -3,and -4) causing disease in human were known, but a fifth serotype was recently discovered in Malaysia [1,2].

Over the last decades, DENV has become the most important arthropod-borne virus affecting humans. Several factors such as rapid urbanization, failure to control mosquitoes and rapid progress in air transportation have contributed to the emergence of endemic dengue in 128 countries in the world [3,4]. In 2009, the World Health Organization (WHO) estimated that 2.5 billion people were living in areas at high risk for infection, among which 50 million were infected annually [1]. However due to weak disease surveillance, low level of reporting of cases and difficulties in diagnosis, the true incidence and burden of dengue are very likely under estimated. Using new modeling approaches, Bhatt et al. estimated that approximately 3.97 billion people were living in areas where DENV is circulating and that almost 400 million cases of dengue occurred every year worldwide [4].

While most infections are asymptomatic or result in a mild febrile illness, the virus is capable of producing life-threatening dengue hemorrhagic fever, dengue shock syndrome, and non-specific complication of systemic diseases (e.g. encephalitis, hepatitis) [1]. The course of dengue illness can be divided in three main phases: the febrile phase, the critical phase and the recovery phase. Severe clinical disease manifestations such as fluid leakage, bleeding and shock occurs during the critical phase which begins around day 4–7 after the onset of fever, when the temperature drops under 38°C, and lasts usually between 48 to 72 hours. During the critical phase, the condition of patients experiencing a severe form of the disease worsens as a result of plasma leakage whereas the condition of patients with a non-severe disease improves.

Direct diagnosis of DENV infection is based on virus isolation, detection of the viral genome by reverse transcription polymerase chain reaction (RT-PCR) or detection of NS1 antigen. Indirect diagnosis using serological methods to detect anti-DENV IgM and IgG is commonly employed, while IgA tests remain less commonly used. The selection of diagnostic methods depends greatly on the time-point of the sample collection during the course of the disease. RT-PCR and virus isolation require a blood sample collected during the early febrile phase of the disease (0–5 days after the onset of fever) [1]. A sample obtained during the early phase is also preferred for NS1 detection, but in patients experiencing a primary infection the NS1 antigen remains detectable for nine days or more after the onset of fever [5,6]. Serological methods can be used later during the course of the disease. IgM and IgA, however, can persist in blood during several weeks or even months after the infection while IgG may persist for decades. Differentiating between an acute and a recent DENV infection by these methods may therefore be challenging. Currently almost all laboratory diagnostic methods require a blood sample that may be difficult to obtain in children, the population which is most commonly affected by dengue in endemic regions, especially in field conditions or outbreak settings. Saliva and urine samples could be used as surrogates for blood as the collection of these body fluids is non-invasive and better accepted by patients, does not require medically-trained staff and the samples are easier to process as they require only limited laboratory facilities.

Diagnosis using urine and saliva samples has already been explored for several other viral infections such as HIV, Hepatitis A, B and C and rubella [7–13]. Urine specimens seem suitable for the diagnosis of infection by West-Nile virus (WNV) and Zika virus (ZIKV)–two other flaviviruses—as viral genome of both viruses can be detected longer in urine than in serum [14,15] and infectious WNV and ZIKV can be recovered from urine [16–18]. Previous studies conducted on a limited number of samples have demonstrated that urine and saliva could be used as an alternative to blood for serological or virological diagnosis of dengue [19–29].

The purpose of this study was to describe the excretion profiles of the anti-DENV antibodies (IgG, IgM and IgA), the NS1 antigen, the viral genome and of the infectious virus in plasma, saliva and urine specimens obtained from a large number of Cambodian children with a confirmed dengue infection. In this study, we aimed to evaluate the possibility of using saliva and urine as alternatives to serum or plasma samples. We also aimed to assess if urine and/or saliva specimens could contribute to predict progression towards a severe form of the disease, as suggested by Chuansumrit *et al*. [21], or if the use of such specimens increases the time window for DENV infection confirmation as suggested by several previous studies [20,22,24].

## Materials and Methods

### Clinical samples

Plasma, saliva and urine samples used in this study were collected in Cambodia in 2013, during the DENFREE (DENgue research Framework for Resisting Epidemics in Europe) study [30]. Samples were prospectively obtained from children hospitalized with clinically-suspected dengue in one of the three hospitals of the Kampong Cham province participating to the DENFREE study. Dengue infection was confirmed in patients through a combination of: viral RNA detection by quantitative RT-PCR (qRT-PCR); isolation of the virus in cell culture; detection of the NS1 protein in the blood; by evidencing a IgM seroconversion in paired plasma and/or a fourfold increase of the antibody titer measured by hemagglutination inhibition assay (HIA) in paired plasma collected at least seven days apart. Disease severity was assessed for each patient according to the 1997 WHO criteria and to the 2009 WHO guidelines [1, 31] using clinical, biological and paraclinical examination data recorded at admission and throughout the entire hospitalization period. Patients were classified into 2 groups: non-severe dengue (dengue fever (DF) according to the 1997 guidelines and DF without warning signs according to the 2009 WHO guidelines) and severe dengue (dengue hemorrhagic fever (DHF) and DSS (dengue shock syndrome) according to the 1997 guidelines and dengue with warning signs as well as severe dengue according to the 2009 WHO recommendations). Urine and saliva were collected daily during hospitalization and then one week, two weeks, three weeks, one month and three months after the discharge from hospital. Plasma specimens were collected at three time-points during hospitalization: on admission, during hospitalization and at the time of hospital discharge. If the patient or guardians gave consent, blood was also collected 30 and 90 days after leaving the hospital.

Urine specimens were collected in 50 ml sterile tubes. Due to the large volume of saliva required for the analyses, the saliva specimens were obtained by direct spitting into 1.8 ml sterile tubes. If possible, children were asked to spit into two separate tubes, an empty one and a pre-filled one with 100µl of viral transport medium (VTM), until minimal volumes of 100 µl and 200 µl were reached in the empty and the pre-filled tubes, respectively. All clinical samples were stored at -80°C prior to testing. In addition to prevent degradation of the RNA and of the virus infectivity, the freezing process was also beneficial to reduce the viscosity of the saliva.

Only patients with well-documented medical records allowing a clear assessment of the disease severity were included in this study.

The clinical specimens obtained from 20 patients hospitalized for a non-dengue febrile illness were also used to assess the specificity of the different diagnostic methods in urine and saliva samples. These controls showed no biological evidences of on-going DENV infection (DENV qRT-PCR negative, NS1 test negative and no anti-DENV antibodies in paired plasma [admission and discharge]).

### Viral genome detection

The viral RNA was extracted from 140 µl of plasma, 280 µl of saliva mixed with VTM and 280 µl of concentrated urine, using the QIAmp Viral RNA Mini kit (Qiagen, Germany). Urine samples were concentrated using the 100K Microsep ultrafiltration device (Pall, USA) to convert an initial volume of 5 ml urine to a final volume of 280 µl of concentrated urine. After extraction, the DENV nucleic acids present in plasma were detected by a serotype-specific quantitative multiplexed real-time RT-PCR (qRT-PCR) as previously described by Hue *et al*. [32]. For urine and saliva, the multiplex qRT-PCR was replaced by a monoplex qRT-PCR as the serotype was already identified in the corresponding plasma. Results were expressed in equivalents to complementary DNA (cDNA) copies per ml. The limit of detection in plasma was 350 copies/ml for DENV-1, 75 copies/ml for DENV-2, 350 copies/ml for DENV-3 and 715 copies/ml for DENV-4. The limit of detection was 200 copies/ml, 50 copies/ml, 200 copies/ml and 350 copies/ml for DENV-1, -2, -3 and -4, respectively, with the qRT-PCR in urine and plasma specimens spiked with quantified synthetic plasmids.

As DENV viremia lasts only a few days after the onset of fever (DAOF), the samples selected for the detection of the viral genome were mainly those collected during hospitalization. Additional urine and saliva specimens collected one to three weeks after discharge from the hospital were also added as some data previously published suggested that the DENV genome could be detected in urine until day 16 after the onset of fever [22].

### Virus isolation in C6/36 cells

C6/36 cells were used in attempt to isolate DENV from urine and saliva samples. Before inoculation, urine samples were dialyzed and concentrated in PBS using the 100K Microsep ultrafiltration system (Pall, USA) and then filtered through 0.2-μm membrane (Nalgene Thermo Scientific, USA). Saliva diluted in VTM as well as urine samples were diluted 1/2 with L15 Leibovitz Medium (Sigma Aldrich, Germany) containing 2% of fetal calf serum (Gibco Life Technology, USA). Final volumes of 300 µl of diluted specimens, or controls, were inoculated into 12-well plates containing 100% confluent C6/36 cells and incubated for 1 hour at 28°C. After incubation, 1.7 ml of medium was added to each well and the plates were incubated at 28°C. After 7 days, cells were harvested and DENV was detected by an immunofluorescence assay using serotype-specific monoclonal antibodies as described previously [33]. For samples that tested negative, two additional passages on C6/36 were performed before concluding that DENV did not replicate.

Our positive controls consisted of urine and saliva specimens obtained from healthy individuals as well as VTM spiked with infectious virus at a final concentration of 3 to 7 log10 cDNA copies/ml. Spiked urine controls were dialyzed and concentrated as for the patient’s urine samples.

### NS1 detection by ELISA

A capture ELISA was used to detect NS1 in plasma, non-concentrated urine and undiluted saliva. In plasma and urine, the NS1 protein was detected by the two-step ELISA method described by Alcon et al. [6] but after replacing polyclonal antibodies by monoclonal antibodies. For the saliva specimens, a one-step capture ELISA was used. All the protocols are detailed in S1 Table. Briefly, in the one-step ELISA developed for NS1 detection in saliva, the sample and the conjugated antibody were incubated together for 2 hours. In the two-step ELISAs (used for plasma and urine specimens), samples were first incubated for one hour and then after a washing step, the conjugated antibody was added and incubated for one hour.

For the patients infected with DENV-1, the NS1 protein was quantified by ELISA using serial two-fold serial dilutions of a solution containing a known concentration of affinity-purified DENV-1 recombinant NS1 protein expressed in HEK293T cells (kindly provided by Dr. Marie Flamand, Institut Pasteur, Paris, France). The standard curve was linear between 0.5 ng/ml and 16 ng/ml for all three NS1 assays. A result was considered positive if the OD was greater than twice the mean OD value measured in 20 negative controls (20 healthy children enrolled during the community study of DENFREE project).

Two positive controls, a weak one and a strong one, and one negative control were used to validate the results of each plate. Inter- and intra-precision of the different ELISAs are shown in S4 and S5 Tables. The samples used for the NS1 protein detection were mainly those collected between DAOF 1 and 10 in hospitalized patients. In addition, 29 urine and 7 saliva specimens collected between DAOF 10 and 16 were tested.

### IgM and IgA detection by MAC-ELISA and AAC-ELISA and IgG detection by indirect ELISA

In-house capture ELISAs were used to detect anti-DENV IgM (MAC-ELISA) and IgA (AAC-ELISA) in plasma, non-concentrated urine and undiluted saliva specimens. For plasma, MAC-ELISA and AAC-ELISA were performed as described previously [33,34]. For urine and saliva samples, minor modifications to MAC-ELISA and AAC-ELISA protocols were adopted. A 100 µl/well format was used for plasma and urine samples, but as only a limited volume of saliva was available, a 50 µl/well format was used for this body fluid. The protocols used are detailed in S2 and S3 Tables. One negative control, one weakly positive control and one strongly positive control were tested in each plate in order to validate each plate of tests. Inter- and intra-precision of the different assays are shown in S4 and S5 Tables.

Results were expressed as ΔODs (OD of the sample incubated in the presence of antigen—OD of the sample incubated in the absence of antigen). A result was considered as positive when the ΔOD was higher than 0.05 for the saliva-based AAC-ELISA and higher than 0.1 for all the other ELISAs. The cut-offs were determined by testing the samples from 20 healthy children who showed no biological evidences of a previous DENV infection (DENV qRT-PCR negative, NS1 ELISA negative and HI titer = 0). Cut-off values are shown in S1 Fig.

In-house indirect ELISAs were used to detect anti-DENV IgG in plasma, non-concentrated urine and undiluted saliva specimens [35]. A 100 µl/well format was used for the plasma and urine-based ELISAs, while a 50 µl/well format was used for the saliva-based ELISA, due to the limited volume of samples available. The protocols used here are described in S6 Table.

Results were expressed as ΔODs. Results were considered as negative when the ΔOD was below 0.2 for the ELISA in plasma (cut-off determined by comparing ELISA results to those obtained by HIA with the same samples) and below 0.1 for the ELISAs in urine and saliva. Cut-off values were determined by testing the samples obtained from 20 healthy children and are described in S1 Fig.

For the evaluation of the antibody detection assays, the samples tested were collected during the hospitalization as well as during the follow-up period after the patient was discharged from the hospital. The panel contained samples collected as early as the day of the onset of fever as well as specimens obtained up to 103 days after the beginning of the disease.

### Statistical analysis

Statistical analysis was performed using STATA version 11.0 (StataCorp, USA). A significance was assigned at P<0.05 for all parameters and were two-sided. The statistical differences between various categorical groups were detected using Chi-squared test. Correlation coefficients between two continuous variables were calculated using Spearman’s rank correlation test. Uncertainty was expressed by 95% confidence intervals (CI95).

In order to identify explanatory variables associated with the detection of the viral genome, the NS1 antigen or the anti-DENV antibodies in the three different fluids, multivariate analysis using a boosted regression tree (BRT) approach was used. BRT is a method combining regression trees with weak individual predictive performances into a single model with higher performance. We used BRT because it is capable of dealing with complex responses, including non-linear relationships and interactions between explanatory variables [36], and we were expecting such complex relationships between our explanatory and response variables. As part of the final model, the BRT assesses the relative importance (RI) of each explanatory variable based on the number of times a variable is used in all trees and its contribution to the final model improvement. A higher RI of a predictor indicates a stronger influence on the response in question (i.e. the detection of each diagnostic marker, in this study). The effect of each explanatory variable on the response can be visualized by the use of partial dependence plots. For each model with a binary response variable, a Receiver-Operating Characteristic (ROC) curve was constructed and the area under the ROC curve (AUC) was calculated. The AUC ranges between 0.5 for random prediction to 1, the higher the AUC the better the model performance. For models with a continuous response variable, the performance of the model was estimated by a correlation coefficient between observed and predicted datasets. This correlation coefficient ranges between 0 (no correlation) and 1 (perfect correlation), with values superior to 0.5 suggesting a high correlation. The analysis was carried out using the “dismo” package (version 1.0–5) [37] and the “gbm” package (version 2.1) [38] under the R statistical environment (R Foundation, Vienna, Austria). A learning rate of 0.001, a bag fraction of 0.5 and a tree complexity of 3 were used. The explanatory variables used are summarized in Table 1. The dengue serotype was not included in the analyses as most of the patients included in this study were infected with a DENV-1 strain during the 2013 epidemic in Cambodia.

**Table 1:** Explanatory variables used in the different BRT models.

Name	Description	Variable type
daof	Number of day between the onset of fever and the time of sampling	Continuous
status	Immune status of the patient determined by HIA	Categorical (Primary infection / Secondary infection / Unknown)
classif	Disease severity classification according to 1997 WHO guideline	Categorical (Non severe / Severe)^a^
logvir	Viral load measured by qRT-PCR (log10 cDNA copies/ml)	Continuous
logNS1	NS1 concentration in plasma (log10 ng/ml)	Continuous
antibodies	Quantity of anti-DENV antibodies in plasma estimated by optical density in ELISA	Continuous

^a^ Non severe disease refers to dengue fever (DF) and severe disease refers to dengue hemorrhagic fever (DHF) and dengue with shock syndrome (DSS)

### Ethical aspects

The children’s legal representatives signed a written consent before the enrolment of the patient. The DENFREE project was approved by the Cambodian National Ethics Committee for Health Research (authorization no. 063NECHR).

## Results

Dengue infection was confirmed in 401 of 574 children hospitalized with clinically-suspected dengue included in the DENFREE study between June and September 2013. In total, 913 plasma, 1555 urine and 1564 saliva samples obtained from 267 patients with confirmed dengue infection and complete records were analyzed. The patients were 3 to 16 years old (median age: 9 years) and 57.3% (153/267) were male. Because of sample volume limitations, all patients may not have been included in each laboratory test evaluation. For each patient, between one to eight urine (median = 4), one to six saliva (median = 3) and one to six plasma (median = 3) samples were collected. Among these samples, a total of 763 matched plasma, urine and saliva samples were obtained from the individual patients at the same time-point during the course of the disease. The number of patients as well as the number and the characteristics of the samples used to evaluate each assay are summarized in Table 2.

**Table 2:** Characteristics of the samples used in each part of the study.

		Plasma	Urine	Saliva
**Complete study**				
	Total number of samples	913	1555	1564
	Total number of patients	267	267	267
**Viral genome detection**				
	Total number of samples	378	442	562
	Total number of patients	144	118	132
	***Immune status***			
	Primary infection	107	139	172
	Secondary infection	185	206	269
	Undetermined^a^	86	97	121
	***Serotype***			
	DENV-1	273	369	401
	DENV-2	40	44	61
	DENV-3	0	0	0
	DENV-4	65	29	100
	qRT-PCR negative	0	0	0
	***Disease classification*** ^***b***^			
	Non severe	210	228	317
	Severe	168	214	245
	***Sample collection***			
	At admission and during hospitalization	378	423	551
	After hospital discharge	0	19	11
**NS1 detection**				
	Total number of samples	533	859	688
	Total number of patients	217	193	197
	***Immune status***			
	Primary infection	157	240	201
	Secondary infection	267	446	342
	Undetermined^a^	109	173	145
	***Serotype***			
	DENV-1	365	592	481
	DENV-2	44	54	58
	DENV-3	9	19	17
	DENV-4	86	147	111
	qRT-PCR negative	29	47	21
	***Disease classification*** ^***b***^			
	Non severe	341	542	477
	Severe	192	317	211
	***Sample collection***			
	At admission and during hospitalization	533	839	687
	After hospital discharge	0	20	1
**Antibodies detection**				
	Total number of samples	876	1,493	1,489
	Total number of patients	255	219	227
	***Immune status***			
	Primary infection	253	469	425
	Secondary infection	450	735	784
	Undetermined^a^	173	289	280
	***Disease classification*** ^***b***^			
	Non severe	586	1,005	1,010
	Severe	290	488	479
	***Sample collection***			
	At admission and during hospitalization	690	1,020	997
	After hospital discharge	186	473	492

^a^Immune status is undetermined when the interval between admission and hospital discharge is < 7 days and the HI titer at the time of hospital discharge is ≤2560

^b^ non-severe dengue corresponds to dengue fever (DF) according to the 1997 guidelines and to DF without warning signs according to the 2009 WHO guidelines. Severe dengue corresponds to dengue hemorrhagic fever (DHF) and DSS (dengue shock syndrome) according to the 1997 guidelines and dengue with warning signs and severe dengue according to the 2009 WHO recommendations.

### Viral genome detection

**Fig 1:**
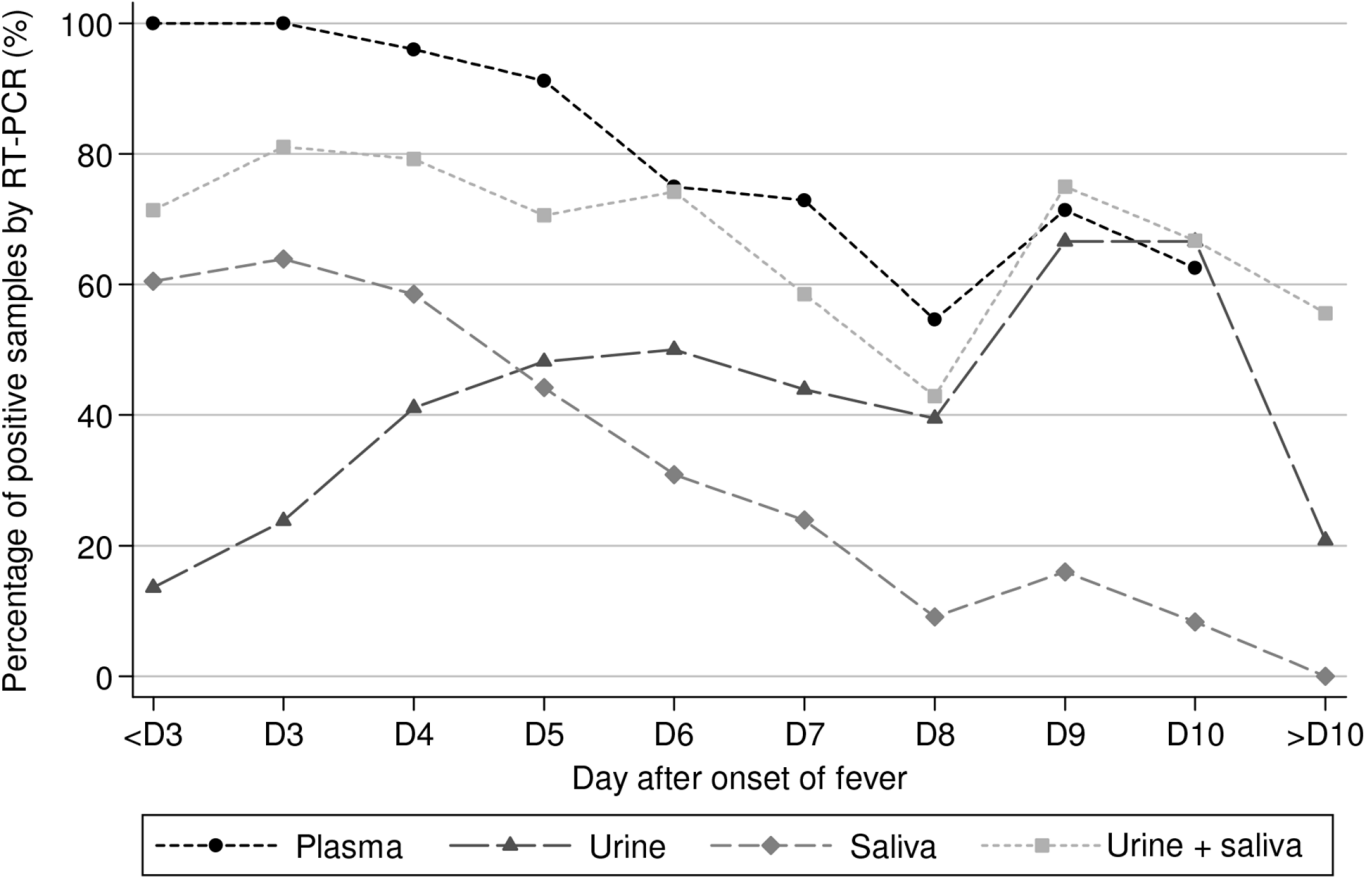
Detection of DENV RNA by qRT-PCR in plasma, urine and saliva specimens. Percentage of plasma, urine and saliva specimens that tested positive for DENV RNA, by day of sampling after onset of fever. (n = 144 patients for plasma, 118 for urine and 144 for saliva).

Overall, 85.4% (323/378, CI95 = [81.5–88.8]) of the plasma specimens obtained from 144 patients with a confirmed acute DENV infection and sampled from the day of onset of fever until the 10^th^ day after fever onset, tested positive. Out of the 442 urine and the 562 saliva samples obtained between the day of fever onset until the 28^th^ day after the onset of fever (DAOF 28) from 118 and 132 patients, respectively, 41.6% (n = 184, CI95 = [37.0–46.4]) and 39% (n = 219, CI95 = [34.9–43.1]) tested positive by qRT-PCR. The qRT-PCR results are presented based on days of sampling after the onset of fever (Fig 1, S7 Table, S9 Table). The proportion of urine samples positive by qRT-PCR increased with time, starting from a minimum of 13.6% during the first 2 days after fever onset to a maximum of 66.6% at DAOF 9–10. At the latest time-points, the proportion of urine samples positive by qRT-PCR was approximately the same as the proportion of RNA-positive plasma specimens (71.4% and 62.5% at DAOF 9–10, respectively). DENV RNA remained detectable in one urine sample at DAOF 16. The detection rate of viral RNA by qRT-PCR in saliva samples followed the decreasing curve of the RNA detection rate observed in the plasma specimens. The highest proportion of saliva samples that tested positive for DENV RNA was observed between the first day and the 4^th^ day of fever (60.5% at DAOF 0–2, 63.9% at DAOF 3 and 58.5% at DAOF 4) whereas plasma samples collected at the same time-points were RNA-positive in 96% to 100% of the cases. The RNA detection rate in saliva decreased thereafter (44.2%, 30.9%, 23.9%, 9.9%, 16% and 8.3% at DAOF 5, 6, 7, 8, 9 and 10, respectively), and all samples tested negative after DAOF 10. By comparison with the percentage of plasma samples positive by qRT-PCR, the sensitivity of qRT-PCR in saliva was on average 46.2% lower, with a minimal difference of 36.1% at DAOF 3 and a maximal difference of 55.4% at DAOF 9.

In total, 57.6% (68/118) of the patients had at least one of their urine samples that tested positive by qRT-PCR and 68.9% of them (92/132) had at least one saliva sample that tested positive.

When only considering the 243 matched plasma, urine and saliva samples (i.e clinical specimens collected from the same patients at the same time-points) obtained between DAOF 1 and DAOF 12, the proportion of specimens that tested positive by qRT-PCR was 90.5% (220/243, CI95 = [86.1–93.9]), 39.9% (97/243, CI95 = [33.7–46.4]) and 49.8% (121/243, CI95 = [43.3–56.3]) for plasma, urine and saliva, respectively (Table 3). Three urine samples that tested positive had a corresponding matched plasma sample that tested negative. These 3 urine samples were all collected at the time of hospital discharge: one at DAOF 6 and the two others at DAOF 10. Testing by qRT-PCR the urine and the plasma specimens led to an increase of the diagnostic sensitivity: 90.5% of sensitivity if the plasma was tested alone, 91.8% when both urine and plasma specimens were tested in parallel. The diagnostic sensitivity increased from 85% for plasma samples alone to 86.7% for the plasma and urine combination with samples collected at DAOF 6–7 and from 70.7% to 75.6% during the second week after fever onset. All subjects whose saliva samples tested positive also had detectable RNA levels in their corresponding plasma samples. If both the urine and the saliva samples collected at the same time-points were screened for DENV-RNA the diagnostic sensitivity increased to 69.1% (168/243, CI95 = [62.9–74.9]). If saliva and urine specimens were tested in parallel instead of testing the plasma, the overall diagnostic sensitivity decreased by 21.4% (by 16.7% if the samples were collected at DAOF 0–1 or at DAOF 6–7, by 22.4% at DAOF 3–4, by 23% at DAOF 4–5, and by 24.4% for samples obtained during the 2^nd^ week after the onset of fever).

**Table 3:** Results of viral genome and NS1 protein detection in saliva, urine and plasma samples collected from the same patients at the same time-points by time of sampling after onset of fever. Percentage of positivity (number of positive samples/total number of samples tested).

		D0-1	D2-3	D4-5	D6-7	W2	W3	W4	W5	W6	M3
**Plasma**	**RNA**	100% (6/6)	100% (49/49)	97.7% (85/87)	85% (51/60)	70.7% (29/41)	NA	NA	NA	NA	NA
	**NS1**	83.3% (10/12)	91.6% (76/83)	79% (83/105)	55.9% (33/59)	29.1% (16/55)	NA	NA	NA	NA	NA
	**IgM**	0% (0/11)	5.5% (7/127)	30.5% (47/154)	75.8% (94/124)	82.2% (83/101)	NA	NA	NA	15.7% (11/70)	1.2% (1/86)
	**IgA**	0% (0/9)	2.3% (2/86)	26.7% (32/120)	71.7% (71/99)	91.6% (76/83)	NA	NA	NA	32.3% (20/62)	7.3% (4/55)
	**IgG**	27.3% (3/11)	12.5% (11/88)	39.7% (50/126)	63.3% (62/98)	84.7% (72/85)	NA	NA	NA	82.1% (55/67)	76.1% (67/88)
**Urine**	**RNA**	16.7% (1/6)	20.4% (10/49)	40.2% (35/87)	55% (33/60)	43.9% (18/41)	NA	NA	NA	NA	NA
	**NS1**	0% (0/12)	10.8% (9/83)	28.6% (30/105)	18.6% (11/59)	0% (0/55)	NA	NA	NA	NA	NA
	**IgA**	0% (0/9)	0% (0/86)	12.5% (15/120)	42.4% (42/99)	72.3% (60/83)	46.8% (29/62)	25.4% (17/67)	11.4% (5/44)	3.2% (2/62)	1.8% (1/55)
	**IgG**	0% (0/11)	3.4% (3/88)	26.2% (33/126)	52% (51/98)	75.3% (64/85)	61.7% (37/60)	50.8% (32/63)	41.9% (18/43)	28.4% (19/67)	12.5% (11/88)
**Saliva**	**RNA**	83.3% (5/6)	75.5% (37/49)	60.9% (53/87)	35% (21/60)	12.2% (5/41)	NA	NA	NA	NA	NA
	**NS1**	16.7% (2/12)	33.7% (28/83)	41% (43/105)	28.8% (17/59)	10.9% (6/55)	NA	NA	NA	NA	NA
	**IgM**	0% (0/11)	6.3% (8/127)	26.6% (41/154)	66.9% (83/124)	75.3% (76/101)	NA	NA	NA	7.1% (5/70)	1.2% (1/86)
	**IgA**	0% (0/9)	0% (0/86)	13.3% (16/120)	49.5% (49/99)	68.7% (57/83)	33.9% (21/62)	13.4% (9/67)	15.9% (7/44)	3.2% (2/62)	0% (0/55)
	**IgG**	9.1% (1/11)	9.1% (8/88)	22.2% (28/126)	52% (51/98)	78.8% (67/85)	80% (48/60)	87.3% (55/63)	79.1% (34/43)	68.6% (46/67)	45.5% (40/88)
**Plasma + urine**	**RNA**	100% (6/6)	100% (49/49)	97.7% (85/87	86.7% (52/60)	75.6% (31/41)	NA	NA	NA	NA	NA
	**NS1**	83.3% (10/12)	91.6% (76/83)	79% (83/105)	55.9% (33/59)	29.1% (16/55)	NA	NA	NA	NA	NA
	**IgA**	0% (0/9)	2.3% (2/86)	28.3% (34/120)	71.7% (71/99)	91.6% (76/83)	NA	NA	NA	32.3% (20/62	9.1% (5/55)
	**IgG**	27.3% (3/11)	12.5% (11/88	44.4% (56/126)	67.4% (66/98)	85.9% (73/85)	NA	NA	NA	82.1% (55/67)	78.4% (69/88)
**Plasma + saliva**	**RNA**	100% (6/6)	100% (49/49)	97.7% (85/87)	85% (51/60)	70.7% (29/41)	NA	NA	NA	NA	NA
	**NS1**	83.3% (10/12)	91.6% (76/83)	79% (83/105)	55.9% (33/59)	29.1% (16/55)	NA	NA	NA	NA	NA
	**IgM**	0% (0/11)	7.1% (9/127)	33.1% (51/154)	79% (98/124)	85.2% (86/101)	NA	NA	NA	17.1% (12/70)	1.2% (1/86)
	**IgA**	0% (0/9)	2.3% (2/86)	27.5% (33/120)	72.7% (72/99)	91.6% (76/83)	NA	NA	NA	32.3% (20/62	7.3% (4/55)
	**IgG**	27.3% (3/11)	15.9% (14/88)	42.9% (54/126)	66.3% (65/98)	85.9% (73/85)	NA	NA	NA	83.6% (56/67)	78.4% (69/88)
**Urine + saliva**	**RNA**	83.3% (5/6)	77.6% (38/49)	74.7% (65/87)	68.3% (41/60)	46.3% (19/41)	NA	NA	NA	NA	NA
	**NS1**	16.7% (2/12)	34.9% (29/83)	49.5% (52/105)	32.2% (19/59)	10.9% (6/55)	NA	NA	NA	NA	NA
	**IgA**	0% (0/9)	0% (0/86)	19.2% (23/120)	56.6% (56/99)	84.3% (70/83)	59.7% (37/62)	29.8% (20/67)	20.5% (9/44)	6.5% (4/62)	1.8% (1/55)
	**IgG**	9.1% (1/11)	11.4% (10/88)	34.9% (44/126)	63.3% (62/98)	82.4% (70/85)	83.3% (50/60)	88.9% (56/63)	81.4% (35/43)	76.1% (51/67)	47.7% (42/88)
**All**	**RNA**	100% (6/6)	100% (49/49)	97.7% (85/87)	86.7% (52/60)	78% (32/41)	NA	NA	NA	NA	NA
	**NS1**	83.3% (10/12)	91.6% (76/83)	79% (83/105)	55.9% (33/59)	29.1% (16/55)	NA	NA	NA	NA	NA
	**IgA**	0% (0/9)	2.3% (2/86)	29.2% (35/120)	71.7% (71/99)	91.6% (76/83)	NA	NA	NA	32.3% (20/62	9.1% (5/55)
	**IgG**	27.3% (3/11)	15.9% (14/88)	46.8% (59/126)	68.4% (67/98)	87.1% (74/85)	NA	NA	NA	83.6% (56/67)	79.6% (70/88)

NA: No sample Available

All clinical specimens obtained from 20 patients hospitalized for a non-dengue febrile illness tested negative.

**Fig 2:**
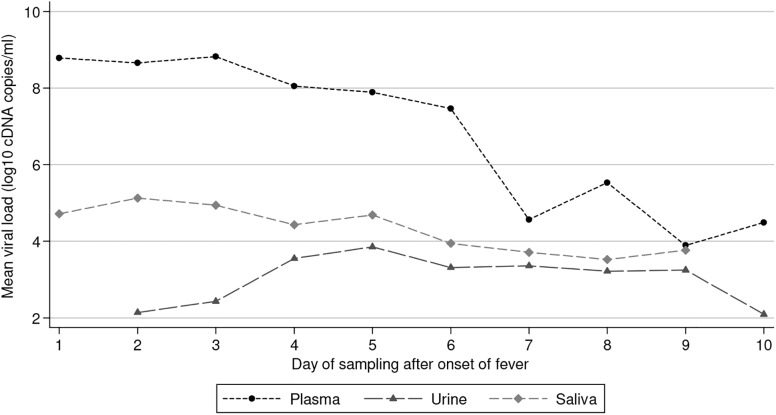
Mean viral load measured by qRT-PCR in plasma, urine and saliva.

On average, 8.2 log10 cDNA copies/ml (min = 2.4 log10 and max = 9.9 log10 cDNA copies/ml) were detected in plasma samples collected between DAOF 0 and 10, 3.5 log 10 cDNA copies/ml (min = 1 log 10 and max = 5.2 log10 cDNA copies/ml) were measured in urine specimens obtained between DAOF 1 and 16, and 4.6 log10 cDNA copies/ml (min = 1.1 log10 and max = 6.1 lon10 cDNA copies/ml) were detected in saliva specimens collected between DAOF 1 and 10. The results from 261, 167 and 164 sequential positive plasma, urine and saliva samples collected between DAOF 1 and 10 and obtained from 103, 57 and 54 patients, respectively, were used to estimate the mean viral load according to the DAOF. The results are presented in Fig 2. Briefly, before the 4^th^ day after the onset of fever, the mean viral load in plasma was at its maximum, at approximately 8.5 log10 cDNA copies/ml. Thereafter, the mean viral load in those samples that tested positive progressively decreased over time. The mean viral load in urine increased from 2.1 to 3.8 log10 cDNA/copies from the 2^nd^ day until the 5^th^ day after onset of fever. Then it decreased very slowly until DAOF 9 before significantly dropping at DAOF 10. Until DAOF 6, the mean viral load in saliva specimens that tested positive by qRT-PCR was approximately 3.5 log10 lower than in plasma. From DAOF 7, the mean viral load in saliva continued to only very slightly decrease and at DAOF 9 reached the same level than in plasma. The viral load measured in saliva correlated with the viral load measured in plasma (Spearman coefficient = 0.51, p-value<0.001) whereas there was no correlation between viral loads in plasma and in urine (p-value = 0.28).

### Virus isolation in C6/36 cells

In total, 15 urine and 15 saliva samples that tested positive by qRT-PCR with a high viral load, i.e. between 3.5 and 5 log10 cDNA copies/ml, were inoculated onto C6/36 mosquito cells. Even after 3 passages, all cell cultures remained negative by immunofluorescence assay. DENV was isolated only from VTM, urine and saliva controls spiked with virus at a concentration equal to or greater than 4 log10 cDNA copies/ml but not when the initial virus concentration was lower. No cell death was observed in the wells inoculated with the three different negative controls i.e., cell culture medium, dialyzed urine samples and saliva samples obtained from healthy donors.

### NS1 detection by capture ELISA

A total of 856 urine and 688 saliva samples obtained from 193 and 197 patients with confirmed dengue were tested. In total, 14.5% (n = 124, CI95 = [12.2–17.0]) of the urine samples and 28.3% (n = 195, CI95 = [25–31.9]) of the saliva samples tested positive by NS1 ELISA. By comparison, 63.4% (338/533, CI95 = [59.2–67.5]) of all plasma samples obtained from hospitalized patients and included in this study tested positive for NS1 (S7 Table, S9 Table).

**Fig 3:**
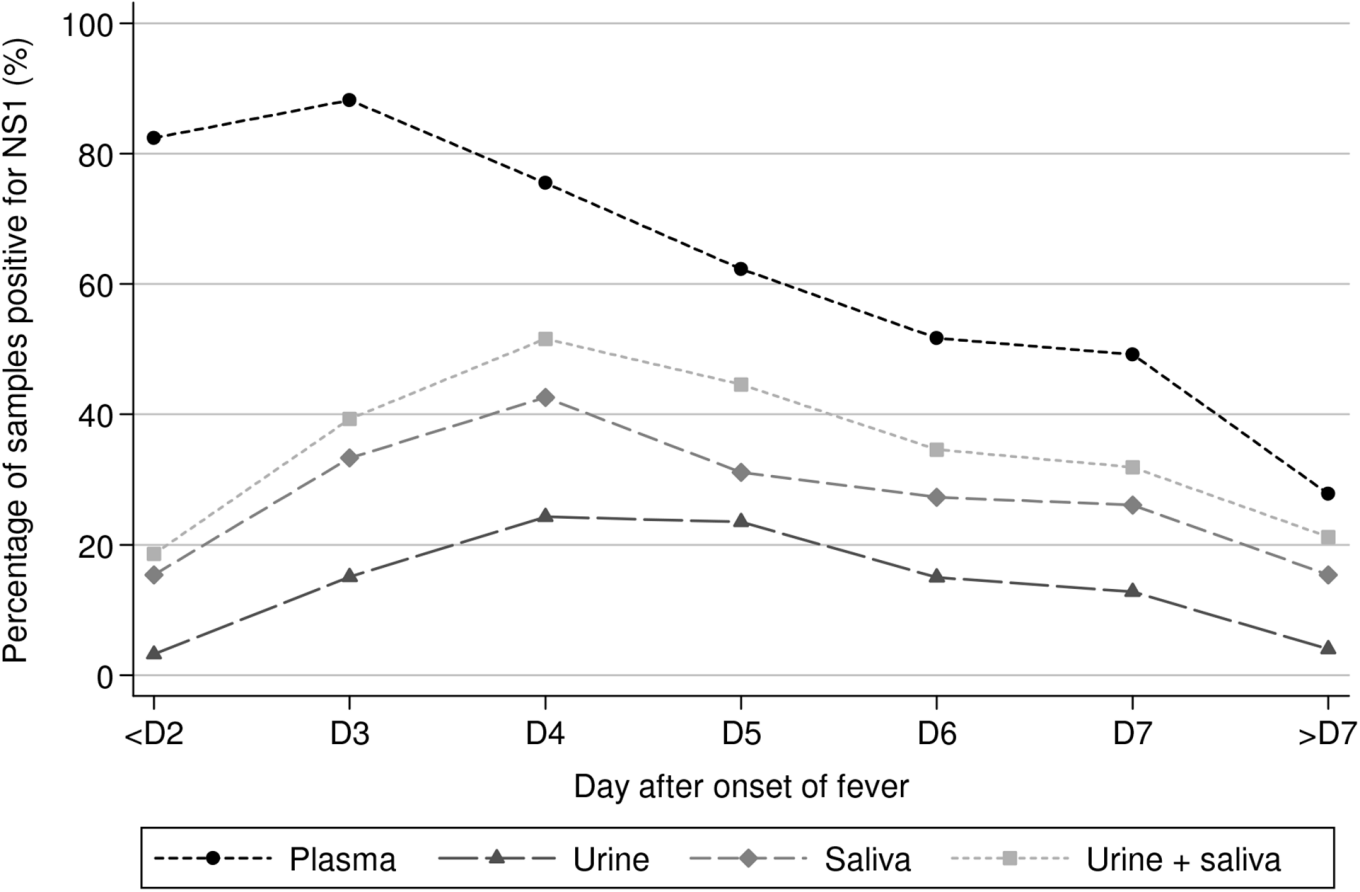
Positivity rates of NS1 protein detection in plasma, urine and saliva. Percentage of samples that tested positive for NS1 protein by capture ELISA in urine, saliva and plasma, by day of sampling after onset of fever (n = 193, 197 and 217 patients with a confirmed dengue infection included for NS1 detection in urine, saliva and plasma, respectively).

The positivity rates of NS1 antigen detection in plasma, urine and saliva samples by day of sampling after the onset of fever are presented in Fig 3 and S7 Table. The NS1 protein was detected in 15.4% of the saliva and in 3.2% of the urine specimens at the beginning of the disease. This percentage increased to reach a maximum of 42.6% in saliva samples and 24.3% in urine specimens at DAOF 4. The proportion of NS1-positive samples then decreased slowly until DAOF 10 and no sample tested positive after that time-point. In plasma, the proportion of samples that tested positive for NS1 was the highest (88.2%) at DAOF 3 and then progressively decreased over time. At DAOF 9, the last time-point at which the plasma specimens were tested, 22.2% of the samples tested positive by NS1 ELISA.

In total, 46.2% (91/197) and 37.8% (73/193) of the patients tested positive in NS1 capture ELISA in at least one of their saliva and urine samples. Among the 91 patients that had at least one NS1-positive saliva sample, the first specimen collected at admission tested positive in 67% (n = 61) of the cases. Among the 73 patients that had at least one of their urine samples that tested positive, only 41.1% (n = 30) had a positive urine sample at the time of hospital admission. Using the results obtained by testing the sequential biological samples collected throughout the hospitalization, the median first day of the NS1 detection in urine was determined for 30 patients at DAOF 5 (mean = 5.1), with a minimum at DAOF 3 and a maximum at DAOF 9. Based on a subset of 17 patients, we were able to establish that the median duration of NS1 antigen detection in the urine of a DENV-infected patient was 1 day (mean = 1.5), with a maximum of 3 days.

The NS1 antigen was detected in 70.9% (202/285), 27.4% (46/168) and 47.7% (73/153) of the plasma, urine and saliva specimens that also tested positive by qRT-PCR. The NS1 ELISA was also positive in 20.9% (9/43), 10.7% (21/196) and 20.7% (42/203) of the plasma, urine and saliva samples that tested negative by qRT-PCR.

NS1 ELISA was used to test a total of 314 plasma, urine and saliva samples obtained from the same patients at the same time-points, between DAOF 1 and DAOF 12. The results were positive in 69.4% (218/314, CI95 = [64.0–74.5]), 15.9% (50/314, CI95 = [12.1–20.4]) and 30.6% (96/314, CI95 = [25.5–36.0]) of the plasma, urine and saliva samples, respectively (Table 3). An overall diagnostic sensitivity of 34.4% (108/314, CI95 = [29.2–39.9]) was obtained by combining the NS1 results of urine and saliva. If saliva and urine samples were tested together instead of plasma, diagnostic sensitivity decreased to 16.7%, 34.9%, 49.5%, 32.2% and 10.9%, at day 0–1, 2–3, 4–5, 6–7 and during the second week after the onset of fever, respectively, while sensitivity in plasma was 83.3%, 91.6%, 79%, 55.9% and 29.1% at the same time-points. Each time a saliva or a urine sample tested positive, the NS1 antigen was also detected in the corresponding plasma specimen. All clinical specimens obtained from 20 patients hospitalized for a non-dengue febrile illness tested negative.

Between DAOF 1 and 10, the average concentration of NS1 protein detected in the clinical specimens was 889 ng/ml (min = 0.8 ng/ml and max = 8 µg/ml) in plasma, 7.2 ng/ml (min = 0.5 ng/ml and max = 60 ng/ml) in urine and 3.8 ng/ml (min = 0.5 ng/ml and max = 41.5 ng/ml) in saliva. The mean NS1 concentration in the plasma specimens increased from 800 ng/ml at DAOF 1–2 to reach a maximal mean concentration of 1.2 µg/ml at DAOF 5–6. It then decreased to reach a concentration of 400 ng/ml at DAOF 8–10 (S2 Fig). The mean NS1 concentration in urine was stable between DAOF3 and 5 (7 ng/ml) and increased at DAOF 6 (14 ng/ml) and DAOF 7 (26 ng/ml) (S2 Fig). No significant variation in NS1 concentration was observed over time in saliva samples (S2 Fig).

### Detection of anti-DENV antibodies

A total of 100 urine specimens were initially tested by MAC-ELISA and since all of them were negative we decided not to include additional samples to the evaluation panel. A total of 1493 and 1483 urine samples were used to evaluate the performances of the IgG and IgA ELISAs in this biological fluid. Out of the 1489 saliva samples available for serological evaluation, 1123, 1395 and 1101 were used for the IgG, IgM and IgA assays, respectively. A total of 766, 678 and 778 plasma samples were tested by indirect IgG ELISA, MAC-ELISA and AAC-ELISA, respectively.

Anti-DENV IgM were detected in 36% (244/678, CI95 = [32.4–39.7]) and 38.1% (531/1395, CI95 = [35.5–40.7]) of all the plasma and saliva samples, respectively, while none of the urine specimens (0/100) tested positive by MAC-ELISA. Overall 37.4% (291/778, CI95 = [34.0–40.9]) of the plasma, 26.8% (397/1483, CI95 = [24.5–29.1]) of the urine samples and 28.6% (315/1101, CI95 = [26.0–31.4]) of the saliva samples tested positive by AAC-ELISA. In total, 54.4% (417/766, CI95 = [50.8–58.0]) of the plasma samples, 38.5% (575/1493, CI95 = [36.0–41.0]) of the urine samples and 52.9% (594/1123, CI95 = [49.9–55.8]) of the saliva specimens tested positive by indirect IgG ELISA.

**Fig 4:**
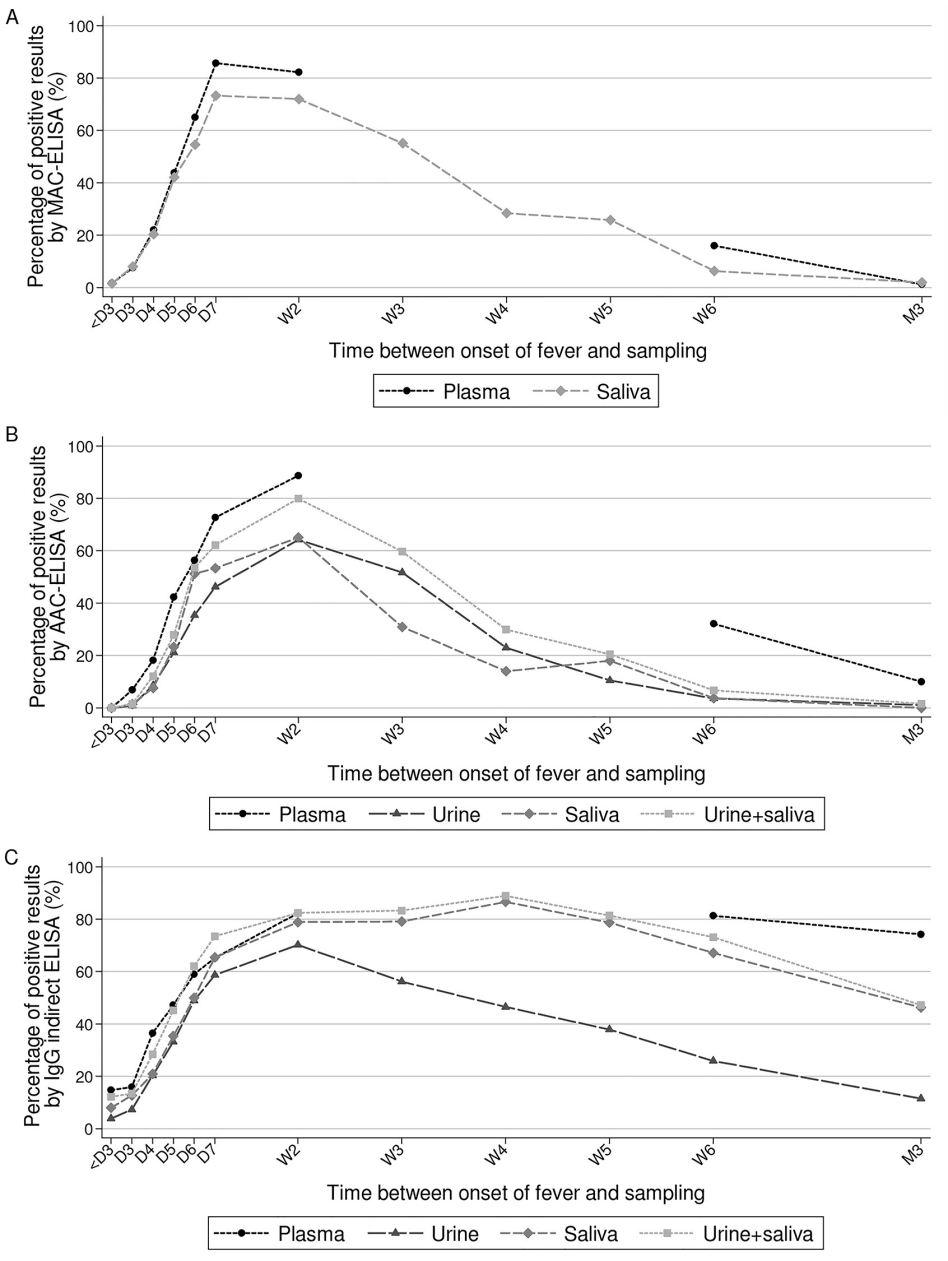
Detection of anti-DENV antibodies in plasma, urine and saliva. Percentage of samples that tested positive by MAC-ELISA (A), AAC-ELISA (B) and IgG indirect ELISA (C) in urine, saliva and plasma samples according to time after onset of fever (D for day, W for week and M for month).

Fig 4 and S7 Table show the percentage of positive results obtained for anti-DENV IgM, IgA and IgG detection in urine, saliva and plasma samples according to DAOF. The positivity rates obtained for IgM, IgA and IgG antibodies detection in the saliva samples were slightly lower than the ones observed for these antibodies in plasma specimens, but both kinetics were in general very similar. MAC-ELISAs sensitivity increased until DAOF 7, to reach 86% and 73% in plasma and saliva, respectively. The sensitivity remained stable during the second week of the disease and then decreased gradually over the following weeks. Six weeks after the onset of the fever, 16% and 6% of the plasma and saliva samples tested positive. At three months, less than 2% of the plasma and saliva samples still contained detectable levels of anti-DENV IgM.

The profiles of the sensitivity kinetic curves for anti-DENV IgA were similar in plasma, urine and saliva specimens. The sensitivity increased until the second week after the onset of fever to reach a maximum of 90% in the plasma and 65% in both the urine and the saliva specimens. The detections rates in urine and saliva then decreased and six weeks after the beginning of the infection only 3% of the urine and saliva samples still tested positive. The anti-DENV IgA antibody was detected in 30% and 10% of the plasma samples collected six weeks and three months after the onset of fever, respectively.

All of the three kinetic curves of the anti-DENV IgG antibodies detection also had a similar overall profile. The proportion of plasma samples that tested positive for IgG quickly increased after the onset of the disease and between the three last collection points the values varied only slightly from 82% at week 2 to 74% at month 3 after fever onset. The percentage of urine samples that tested positive for IgG during the first week was on average 11% lower compared to the percentage of plasma samples that were IgG-positive. Globally both kinetic curves followed the same trend. After a peak at 70.2% during the second week of the disease, the curve demonstrated a regular decrease. In total, 25.9% of the samples tested positive one month after the infection and only 10% after three months. The IgG detection rate in saliva increased continuously from the 1^st^ day of fever until the second week when it reached a plateau around 80%. The sensitivity of the IgG test in saliva samples during the first two weeks of the disease was on average 7% lower compared to the sensitivity of the assay in plasma specimens. From week 5, the detection rate began to decrease and three months after the infection, 46% of the saliva samples remained positive for anti-DENV IgG.

A total of 514 matched plasma, urine and saliva samples collected between DAOF 1 and DAOF 103 were tested by AAC-ELISA. The results were positive in 39.9% (205/514, CI95 = [35.6–44.3]), 23.3% (120/514, CI95 = [19.8–27.2]) and 24.1% (124/514, CI95 = [20.5–28.1]) of the plasma, urine and saliva samples, respectively (Table 3). Concurrent testing of urine and saliva specimens by AAC-ELISA increased sensitivity by 6.7% and 5.9% compared to testing the urine or saliva samples alone and resulted in an overall decrease of the sensitivity to detect anti-DENV IgA by 10%, compared to AAC-ELISA in plasma (decrease by 2.3% for samples collected at day 2–3, by 7.5% at day 4–5, by 15.1% at day 6–7, by 7.3% during the 2^nd^ week, by 25.8% six week after and by 5.5% three months after the onset of fever).

A total of 563 plasma, urine and saliva samples obtained from the same patients at the same time-points, between DAOF 1 and DAOF 103, were tested by IgG indirect ELISA. Anti-DENV IgG were detected in 56.8% (320/563, CI95 = [52.6–61.0]), 32.1% (181/563, CI95 = [28.3–36.2]) and 42.8% (241/563, CI95 = [38.7–47.0]) of the plasma, urine and saliva samples, respectively (Table 3). An overall detection rate of 49.7% (280/563, CI95 = [45.5–53.9]) was obtained by combining the IgG results of paired urine and saliva specimens. The difference between the sensitivity of the IgG test in plasma and in urine and saliva samples varied from a minimum of 9.1% (DAOF 2–3) and 3.4% (DAOF 2–3) to a maximum of 63.6% (month 3) and 30.6% (month 3), respectively. The difference between the sensitivity of the assay in plasma and the one obtained by combining results of paired urine and saliva was below 10% at all time-points (minimal difference: 0% at DAOF 6–7; maximal difference: 7.5% at DAOF 4–5), except at DAOF 0–1 and 3 months after the onset of fever when the difference reached 18.2% and 28.4%, respectively.

### BRT analyses

**Fig 5:**
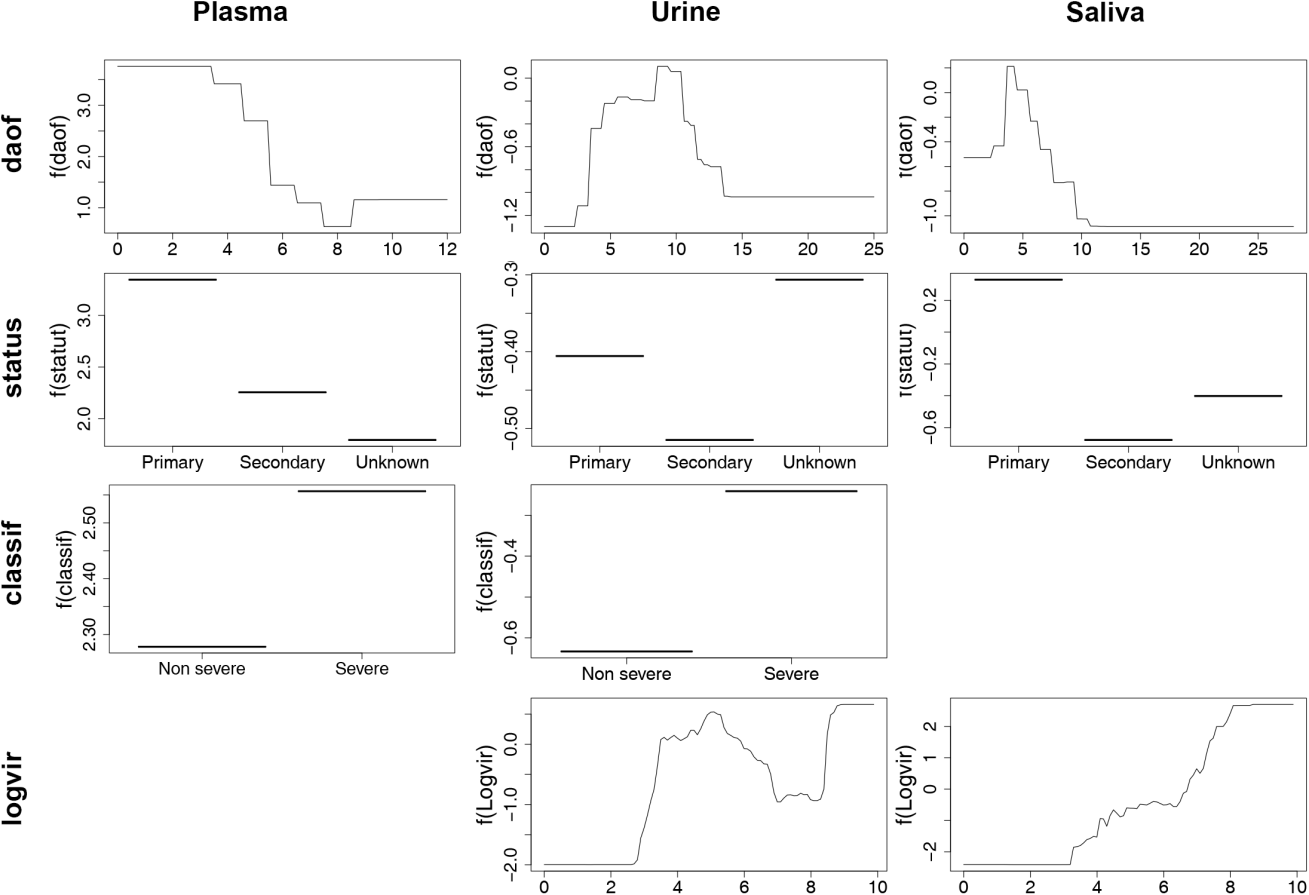
Partial dependence plots for the most influential variables explaining viral genome detection in plasma, urine and saliva.

The BRT analyses demonstrated that the three explanatory variables (“daof”, “status” and “classif”) used in the study had a significant effect on the detection of DENV genome in plasma by qRT-PCR. The variable “daof” (number of days after the onset of fever) had the higher relative importance (RI = 57.4%), followed by the immunological status (primary or secondary infection) (RI = 25.8%) and then by the severity of the disease (RI = 16.8%) (Table 4). The partial dependence plots suggested that there was a higher probability of detecting DENV-RNA in the plasma specimens obtained during the early febrile phase of the infection, in the samples obtained from patients with a primary infection and in patients presenting with a severe form of the disease (Fig 5). Models with four variables (“daof”, “status”, “classif” and “logvir” [viral load in plasma]) were applied for the analysis of the qRT-PCR results in saliva and urine samples. The variable “logvir” was the main explanatory variable (RI saliva = 64.2% and RI urine = 52.6%) in both models, followed by “daof” (RI saliva = 18.5% and RI urine = 26.8%) and “status” (RI saliva = 14.1% and RI urine = 14.4%) (Table 4). The model applied for saliva specimens demonstrated a positive association between the RNA load measured in plasma and the detection of the dengue genome in saliva. With the model used for urine specimens, a more complex relationship with two maxima was observed: the first one for a viral load between 4 and 6 log10 cDNA copies/ml and the second one for a viral load greater than 8 log10 cDNA copies/ml. The detection of the virus genome in saliva and urine samples was better during primary infections (Fig 5). The RI for the variable “classif” was only 3.2% and 6.2% in saliva and urine specimens, respectively (Table 4). The partial dependence plots suggested a slightly better detection of DENV-RNA in the urine of patients experiencing a severe form of the disease (Fig 5).

**Table 4:** Results of the Boosted Regression Trees analysis for the detection of viral genome in plasma, urine and saliva.

Sample	Mean Relative Importance (Standard deviation)				Mean AUC* (Standard deviation)
	daof	status	classif	logvir	
Plasma	57.36 (0.30)	25.80 (0.25)	16.84 (0.26)	-	0.853 (0.007)
Urine	26.79 (0.22)	14.44 (0.19)	6.18 (0.15)	52.60 (0.31)	0.720 (0.008)
Saliva	18.49 (0.20)	14.05 (0.12)	3.22 (0.10)	64.24 (0.26)	0.820 (0.005)

daof: number of days between the onset of the fever and the time of sampling

status: immune status of the patient (primary versus secondary infection) determined by HIA

classif: disease classification according to the 1997 and the 2009 WHO guidelines

logvir: viral load measured by qRT-PCR (log10 cDNA copies/ml)

*Estimate of the model performance

**Fig 6:**
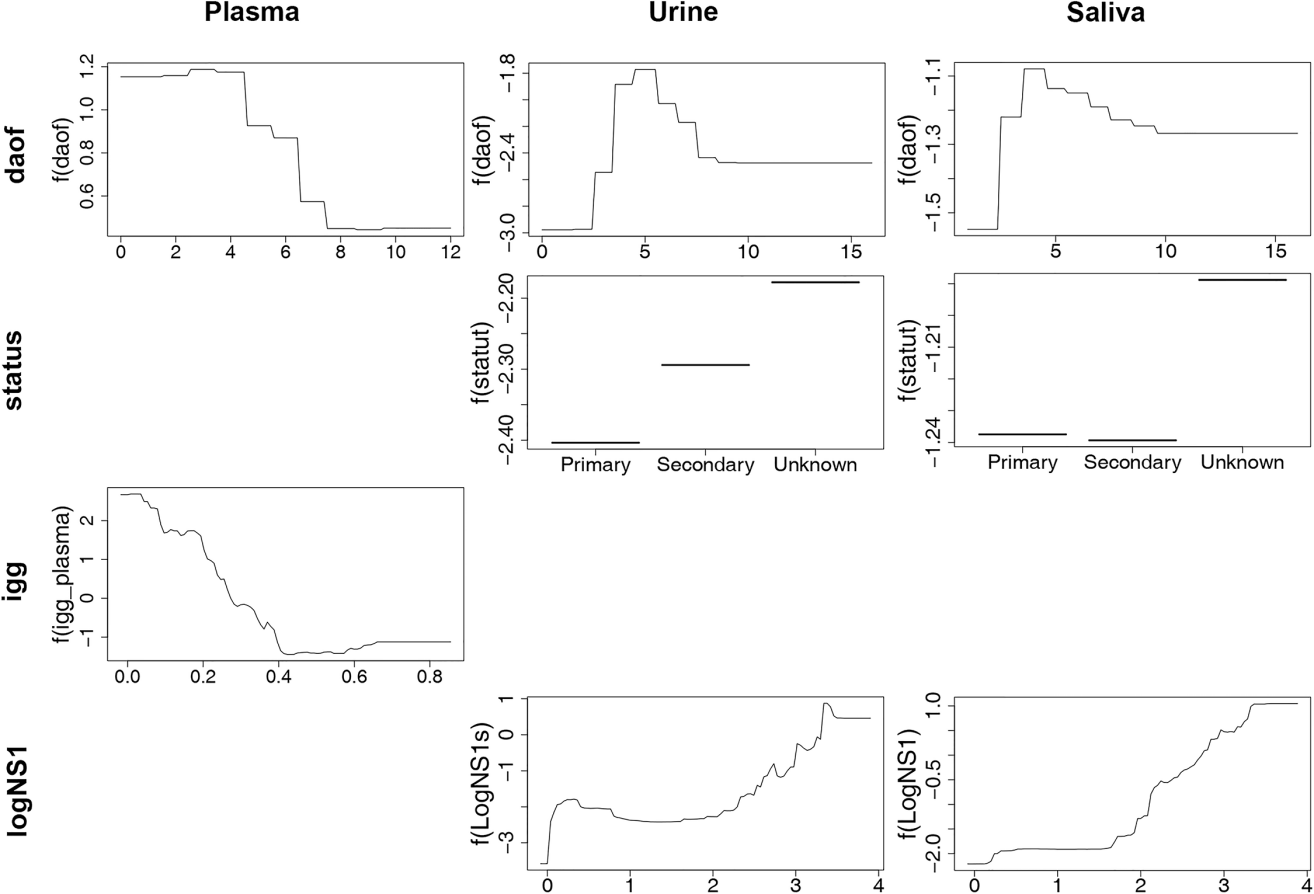
Partial dependence plots for the most influential variables explaining NS1 antigen detection in plasma, urine and saliva.

The best BRT model for NS1 detection in plasma was obtained by using the variables “daof”, “classif” and by replacing the variable “status” by the continuous variable “IgG” (level of IgG antibodies in the plasma specimens estimated by the ELISA OD value). The relative importance of the variable “IgG” was 89.3% and the partial dependence plot demonstrated a nearly linear decreasing association with the detection of NS1 protein (Table 5, Fig 6). The variables “daof” and “classif” had a RI of 8.0% and 2.7%, respectively. The severity of the disease (variable “classif”) can be considered as having a negligible effect on the detection of the NS1 protein in the plasma samples. The BRT models used for NS1 antigen detection in saliva and urine specimens were built with the variables “daof”, “status”, “classif” and “logNS1” (NS1 protein concentration in plasma). The higher RI in both models was obtained for the continuous variable “logNS1” (RI = 67.3% and 64.6% for the saliva and urine models, respectively) (Table 5). The partial dependence plots demonstrated a positive association between NS1 protein concentration in plasma samples and detection of NS1 antigen in saliva and urine specimens (Fig 6). The second main explanatory variable identified was “daof” (RI = 20.9% and 25.4% for the saliva and the urine models, respectively) with an optimal detection of the NS1 protein being between the 3^rd^ and the 8^th^ day after the onset of fever, in both the saliva and the urine samples (Table 5, Fig 6). The contribution to the models of the variables “status” and “classif” was very low (Table 5).

**Table 5:** Results of the Boosted Regression Trees analysis for the detection of NS1 antigen in plasma, urine and saliva.

Sample	Mean Relative Importance (Standard deviation)					Mean AUC*(Standard deviation)
	daof	status	classif	IgG	logNS1	
Plasma	52.59 (0.19)	43.36 (0.30)	4.04 (0.23)	-	-	0.826 (0.004)
Plasma	8.02 (0.16)	-	2.71 (0.08)	89.27 (0.20)	-	0.895 (0.003)
Urine	25.38 (0.29)	7.67 (0.28)	2.32 (0.15)	-	64.63 (0.34)	0.751 (0.006)
Saliva	20.94 (0.75)	9.93 (0.50)	1.83 (0.24)	-	67.30 (0.13)	0.730 (0.004)

daof: number of day between onset of fever and sampling

status: immune status of the patient determined by HIA

classif: disease classification according to the 1997 and the 2009 WHO guidelines

IgG: anti-DENV IgG quantity in plasma estimated by optical density in indirect ELISA

logNS1: NS1 concentration in plasma (log10 ng/ml)

*Estimate of the model performance

**Fig 7:**
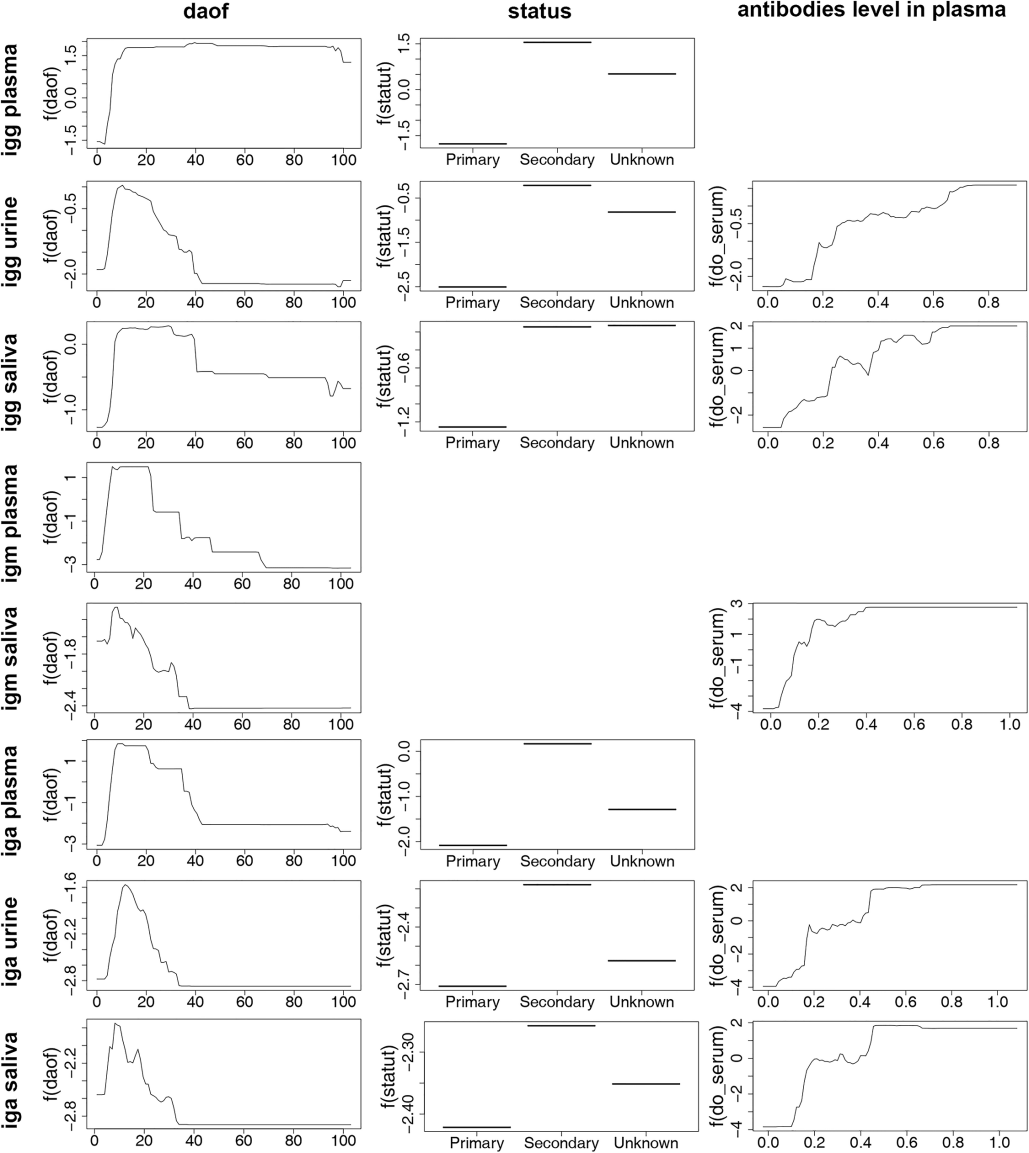
Partial dependence plots for the most influential variables explaining anti-DENV antibodies detection in plasma, urine and saliva.

In all of the BRT models generated for anti-DENV antibodies detection in the three different body fluids, the impact of the explanatory variable “classif” (severe versus non-severe dengue) was negligible. The detection of IgG in plasma specimens was explained by the variables “daof” and “status” with a close relative importance (47.2% and 49.3%, respectively) (Table 6). The partial dependence plots showed that the detection of IgG in plasma was associated with secondary infection and that IgG antibodies were detected from one week until the end of the follow-up period (Fig 7). For the detection of IgM in plasma specimens, the variable “daof” appeared to be the only explanatory variable and was associated with a RI of 95.4%. The RIs of the variables “status” and “classif” were negligible at 3.3% and 1.4% (Table 6). The detection of anti-DENV IgA antibodies in plasma samples was mainly explained by the variable “daof” (RI = 69.8%) and was the highest between one and four weeks after the onset of fever (Table 6, Fig 7). The immune status of the patient (secondary infection) also contributed to explain the ability to detect IgA antibodies (RI = 26.2%) (Table 6, Fig 7). The detection of antibodies in saliva samples was linked mainly to the level of the corresponding class of immunoglobulin in plasma (IgG detection: RI = 50.8%; IgM detection: RI = 68.5%; IgA detection: RI = 58.4%). Similarly, the detection of IgA antibody in urine samples correlated with the titer of IgA in plasma (RI = 60.2%). The relative importance of plasmatic IgG level appeared to be only 20.3% for the detection of IgG in urine specimens. The main variables associated with the presence of IgG in urine samples were the immunological status (RI = 42.1% in secondary infections) and the date of sampling (RI = 36%) (Table 6, Fig 7).

**Table 6:** Results of the Boosted Regression Trees analysis for the detection of anti-DENV antibodies in plasma, urine and saliva.

Antibody	Sample	Mean Relative Importance (Standard deviation)				Mean AUC*(Standard deviation)
		daof	status	classif	antibodies	
IgG	Plasma	47.26 (0.10)	49.31 (0.12)	3.43 (0.06)	-	0.910 (0.002)
	Urine	36.00 (0.12)	42.05 (0.18)	1.66 (0.03)	20.29 (0.16)	0.895 (0.001)
	Saliva	23.49 (0.14)	24.42 (0.14)	1.33 (0.04)	50.76 (0.23)	0.908 (0.002)
IgM	Plasma	95.37 (0.28)	3.26 (0.27)	1.37 (0.11)	-	0.883 (0.003)
	Saliva	26.49 (0.13)	4.62 (0.08)	0.39 (0.03)	68.51 (0.17)	0.878 (0.002)
IgA	Plasma	69.76 (0.20)	26.24 (0.09)	4.00 (0.17)	-	0.907 (0.002)
	Urine	23.68 (0.03)	15.59 (0.10)	0.55 (0.07)	60.18 (0.10)	0.904 (0.002)
	Saliva	26.94 (0.11)	11.54 (0.06)	3.08 (0.05)	58.44 (0.135)	0.899 (0.002)

daof: number of days between the onset of the fever and the day of sampling

status: immune status of the patient determined by HIA (primary versus secondary infection)

classif: severity of the disease according to the 1997 and the 2009 WHO guidelines

antibodies: level of the corresponding immunoglobulin isotype in the plasma estimated by the measure by ELISA of the optical density

*Estimate of the model performance

Two BRT models were used to identify factors influencing NS1 concentration (NS1 model, variable “logns1”) and the RNA load (RNA model, variable “logvir”) in plasma samples during the acute febrile phase of the disease (DAOF 0–5). Both models were built with the explanatory variables “daof”, “classif” and “igg”. In both models the higher RI was obtained for the variable “igg” (RI = 85.2% and 77.3% for the NS1 and the viral RNA models, respectively), followed by the variable “daof” (RI = 8.5% and 20.9% for the NS1 and the viral RNA models, respectively) and the variable “classif” (RI = 6.3% and 1.8% for the NS1 and viral RNA models, respectively) (S8 Table). The partial dependence plot analysis demonstrated that higher NS1 concentrations and RNA loads were associated with very low level of anti-DENV IgG (OD<0.15) (S3 Fig).

## Discussion

One of the objectives of this study was to assess the possibility to use saliva or urine specimens instead of blood for the diagnosis of dengue infections in specific situations. The evaluation included the assays most routinely used for dengue diagnosis: viral genome detection by qRT-PCR, virus isolation in mosquito cell lines, NS1 antigen and anti-DENV antibody detection by ELISA methods. In order to generate the strongest data possible, a large number of samples obtained from children hospitalized in Cambodia for dengue of varying degrees of severity were tested. This is the first study in which plasma, urine and saliva obtained from the same patients, at the same time-points, were tested in parallel and the results compared to estimate the performances of the various dengue diagnostic assays.

Saliva and urine specimens are obtained through non-invasive procedures that require the patient’s participation. If devices for urine sampling in infants exist and if saliva can be collected with small specific swabs, one may consider that in the youngest children the collection of capillary blood on filter paper after a finger-prick could be a better alternative to venipuncture than urine or saliva sampling.

This is the first study that aimed to detect the DENV genome in such a large number of saliva samples and to comprehensively describe the kinetics of DENV genome detection in this body fluid. Previously, Anders *et al*. attempted to detect the DENV genome in saliva swab specimens obtained from six patients with confirmed DENV infection but all samples tested negative [26]. The DENV genome was successfully detected from saliva samples in only few instances and described in some cases reports [20,23] as well as in one very recent study performed on only 14 patients [19]. We demonstrated here that, as in the plasma, the sensitivity of the genome detection in saliva specimens was the highest during the early acute phase of the infection (i.e., 63.9% at day 3 post-fever; approximately 60% during the four first days of the disease) and then decreased over time before the RNA became undetectable 10 days after the onset of fever. The curves of the sensitivity of the RNA detection in plasma and saliva specimens had very similar profiles over time. Nevertheless, the sensitivity of the qRT-PCR in saliva was on average 40% lower than in plasma when the specimens were tested between DAOF0 and DAOF8. The BRT analysis indicated that the higher the RNA load in plasma, the higher the probability of detecting the viral RNA in saliva samples was.

Some authors showed in a limited number of patients and samples that the DENV genome could be detected in urine specimens [19,20,22–24]. Hirayama *et al*. described the results obtained with 77 urine samples collected from 53 patients and described DENV genome excretion kinetics similar to the one we report here, i.e. a delayed excretion of the viral genome in urine by comparison to blood [22]. In some cases, the DENV genome was still detectable in urine samples while the serum already turned negative by qRT-PCR [20,22,24]. In our study, we observed this pattern in three patients whose plasma collected at the time of hospital discharge (up to10 days after onset of fever) tested negative, whereas their urine collected at the same time was positive. The sensitivity of the confirmation of the dengue infection by viral genome detection improved from 79.6% to 82.6% when qRT-PCR was performed in both urine and plasma samples collected after the 5^th^ day of fever, compared to qRT-PCR in plasma alone. Nevertheless, even after DAOF 5, plasma remained the best biological fluid to use for dengue diagnosis. Indeed, out of the 116 pairs of urine and plasma samples collected during the early convalescent phase of the disease from the same patients, 63.8% were concordant (i.e., both urine and plasma samples tested positive or both urine and plasma samples tested negative). Only three pairs demonstrated discordant results with an advantage for urine over plasma whereas 39 pairs gave discordant results in favor of plasma (i.e. the urine sample tested negative whereas the corresponding plasma sample tested positive). After discharge from the hospital, the urine sample of three other patients still tested positive (two patients at DAOF 13 and one patient at DAOF 16) but the comparison with plasma was not possible as blood samples were unavailable for these time points. All patients whose saliva samples tested positive also had detectable levels of RNA in the plasma samples collected at the same time-points. Thus, testing saliva in addition to the plasma samples did not significantly improve diagnostic sensitivity. When it is not possible to get blood samples, testing concurrently both urine and saliva specimens by qRT-PCR could offer an interesting alternative as the overall diagnostic sensitivity was 76.1% during the acute phase of the disease (DAOF 0–5) and 59.4% during the early convalescent phase (DAOF 6–12). Alternatively, the analysis of sequential urine or saliva specimens could also provide, in specific situations, some acceptable results as 57% of the patients had at least one positive urine sample and almost 69% of them had a least one saliva sample that tested positive in the course of the disease.

To date, DENV has never been isolated from urine or saliva. Two unsuccessful attempts to isolate the DENV from urine and one attempt to isolate the virus from saliva were previously reported by Hirayama *et al*. and Korhonen *et al*. [19,22]. The low RNA loads in urine and saliva observed in our study could provide one explanation to the inability to isolate the virus after inoculation of the samples onto C6/36 or Vero E6 cells [39]. Nevertheless, the absence of infectious DENV particles from urine and saliva specimens will be attested only after unsuccessful inoculation of the RNA-positive samples into mosquitoes, as this method is recognized as the most sensitive for DENV isolation [40]. Direct toxicity on C6/36 or Vero E6 cells of some urine and saliva components could also explain the inability to isolate the virus from those samples. In our study, urine samples were dialyzed before inoculation in order to eliminate potentially toxic components and saliva samples were inoculated after dilution with culture media to avoid excessive direct toxicity. Furthermore, the controls included in the series of culture plates provided evidence that a premature cell death was not responsible for the absence of virus detection. It rather appeared that a low viral load was at the origin of an absence of detection of infectious virus in saliva and urine specimens. But it is also possible that the DENV RNA detected by qRT-PCR in urine was “free” viral RNA that was able to go through the glomeruli while virus particles, especially those embedded in large immune complexes, were stopped.

The detection of NS1 protein in saliva and/or urine samples has already been reported in three studies bearing on a limited number of patients [19,21,26] and recently in a larger study including urine samples from 96 patients [41]. Here, we demonstrate that the sensitivity of NS1 detection in saliva and urine samples follow a similar trend over time. Diagnostic sensitivity increased progressively until DAOF 4 to reach a maximum of 25% in urine and 40% in saliva samples, and then decreased until NS1 became undetectable, ten days after fever onset. The sensitivity NS1 antigen detection in urine and saliva samples observed in our study was lower than the one reported previously. Anders *et al*. reported 64.7% (55/85) of saliva samples positive for NS1 protein when testing patients with a positive NS1 antigenemia [26]. In our study, only 25.8% (99/383) of the paired plasma and saliva samples tested both positive, at the same time-point. Chuansumrit *et al*., Korhonen *et al*. and Saito *et al*. previously evaluated the sensitivity of NS1 detection in urine using the same commercial ELISA kit. Chuansumrit *et al*. and Korhonen *et al* reported sensitivities of 65.6% and 54.2%, respectively [19,21]. Saito *et al*. described a positive detection rate of NS1 ranging from 13% to 43% according to days after disease onset. The highest detection rate was obtained on days 6–10 after disease onset [41]. As observed for the RNA load, NS1 protein concentrations measured in urine and saliva samples were much lower than those measured in plasma specimens. The multivariate analysis using BRT identified the NS1 concentration in plasma as the main factor explaining the possibility of detecting the viral protein in urine and saliva. Korhonen *et al*. also showed that the NS1 concentration in urine positively correlated with the concentration in urine of the total proteins [19]. In their study, Chuansumrit *et al* reported a higher NS1 detection rate in urine samples obtained from patients with DHF than in patients presenting with a mild DF [21]. Our statistical analysis performed in a large patient series did not evidence a higher probability of detecting NS1 in the urine of patients experiencing a severe form of the disease (DHF and DSS) than in those presenting with a mild infection. We decided not to concentrate urine specimens before testing for NS1 in order to keep the ELISA protocol as easy and cheap as possible to meet the “real-life” conditions of most endemic countries which are also often developing countries. Saito *et al*. investigated the benefit of urine concentration prior to NS1 capture by ELISA with 37 paired concentrated and non-concentrated urine samples. Concentration allowed the detection of NS1 antigen in three samples that tested negative before concentration [41]. For well-equipped laboratories that may be willing to use urine as a replacement to blood for DENV infection confirmation, it would be important to further develop of a fast and simple method for NS1 protein concentration.

Our study also shows that 2/3 of the plasma samples that tested positive by qRT-PCR also tested positive for NS1 detection; that half of the saliva samples that tested positive by qRT-PCR also tested positive by NS1 capture ELISA, but that NS1 was detected in less than 30% of the urine samples that tested positive by qRT-PCR. The discrepancy between the RNA and NS1 detection in urine samples obtained from patients with detectable viremia suggests that these two biological markers of dengue infection are potentially released in urine through different mechanisms that remain to be clarified.

Similarly to Vasquez *et al*., we were unable to detect anti-DENV IgM in urine specimens [25]. The sensitivity of the IgM and the IgG assays in saliva was close to those obtained in plasma samples. The sensitivity of the IgA serology in both urine and saliva was 65% at the peak point when it was almost 90% in plasma. This is in agreement with the data previously described by Vasquez *et al*. as well as by Balmaseda *et al*. [25,27,42]. Multivariate analysis demonstrated that the probability of detecting antibodies in saliva specimens depended essentially on antibodies titers in the plasma. Cuzzubbo *et al*. reported previously that salivary IgG levels correlated well with serum HI titer [43]. This study also showed that IgG detection in saliva could be used to distinguish between primary and secondary DENV infection. Our BRT analysis confirms this result. Balmaseda *et al*. investigated the use of IgG and IgM detection in saliva to evaluate DENV infection incidence during a serological survey and demonstrated that anti-DENV IgG detection in saliva was a good tool to measure the incidence of dengue in a community cohort [27]. The anti-DENV IgG antibody urinary excretion curve follows the same trend than that of IgG in plasma during the first week of the disease. Subsequently, urinary excretion of IgG decreases over time while it remains stable in plasma. Three months after disease onset, 76.1% of the patients had detectable anti-DENV IgG in their plasma whereas only 12.5% of them still tested positive in their urine. The present study is the first to document the kinetics of anti-DENV antibodies until the third month after fever onset. Multivariate analysis showed that the detection of anti-DENV IgG in urine was not primarily associated with antibody titers in plasma but with the immune status of the patient and with the time of sampling after the onset of the disease. Antibodies are high-molecular-weight (HMW) proteins. In physiologic conditions, these proteins are almost completely restricted from filtration by the glomerular barrier and only a very small quantity of it can normally be detected in urine. The presence of macromolecules such as IgG and IgA in urine could reflect an alteration of the glomerular barrier, with or without tubular cells impairment. IgM is a pentameric immunoglobulin of very high molecular weight (950 kDa) and its detection in urine would reflect a severe glomerular injury [44]. This probably explains why IgM were not detected in the patients included in this study as none of them had any record of severe renal impairment during the course of their illness.

In this study, as in most of those reported previously, saliva specimens were collected by direct spitting. Only Anders *et al*. collected saliva using swabs and reported results significantly different from ours [26]. These authors were unable to detect DENV genome by RT-PCR but obtained a higher sensitivity for NS1 (i.e., 64.7%). Michishige *et al*. demonstrated that saliva samples collected by different methods of sampling were not equivalent in terms of total proteins and secretory IgA concentrations [45]. These different methods of saliva sampling should be compared in order to better define which one is the most suitable to perform each test.

The early identification of an ongoing DENV infection and a rapid implementation of specific clinical management procedures are key to ensure the best clinical outcome [1]. The current lack of simple and reliable prognostic marker to predict the risk of evolution of the patient towards a severe form of the disease makes the clinical management of the DENV infections extremely challenging, especially during epidemics and in countries with weak health systems. Among all the parameters evaluated within this study, the NS1 protein would theoretically be the ideal candidate for early diagnosis as this antigen is a marker of acute dengue infection that is easy and fast to detect. Anti-DENV antibodies usually appear only after the patient has already progressed toward the critical phase of infection. Conflicting results have been reported on a possible association between the concentration of free NS1 circulating in plasma and the disease severity. Although several studies reported a positive association [46,47], other studies have not [48–50]. In our study, which included a large number of patients experiencing primary and secondary infections, the multivariate analysis using BRT suggested that disease severity does not influence NS1 concentration in plasma during the acute phase of the disease but that plasmatic titers of anti-DENV IgG was the most important factor to explain free NS1 protein concentration in blood. Indeed, undetectable or very low levels of anti-DENV IgG antibodies were associated with higher concentrations of free NS1, the only fraction that is detected by the assays, while the NS1 fraction that is trapped into the immune-complexes is not captured in the diagnostic tests.

A potential association between the DENV-RNA load measured during the acute phase of the disease and disease severity has also been suggested, but the scientific literature reports conflicting findings [46,48,51,52]. Our BRT analysis suggests that the severity of infection was not associated to the RNA load in plasma during the acute phase of the disease (DAOF 0–5). The level of anti-DENV IgG was the main parameter explaining the RNA load in the plasma specimens during the acute phase of the disease, with an inverse relationship like the one observed for NS1. The present report is the first to investigate the relationship between the NS1 concentration and the RNA load in plasma and the severity of the disease by using a multivariate analysis that included the anti-DENV IgG level. These findings require to be confirmed during other epidemics involving more patients and other infecting serotypes as this factor may affect both the RNA load and the NS1 concentration [53,51].

Our study shows that urine and saliva could be considered for the diagnosis of dengue infection in some situations when optimal sensitivity is not necessarily required. These two body fluids provide different type of information. The saliva essentially mirrors what happens in the blood, which is not surprising as approximately 2.5% of the whole saliva is composed of gingival fluid, a protein-rich serum transudate, therefore reflecting the protein pattern in the serum. Antibodies titers in saliva are thus a few orders of magnitude lower compared to those in serum [54]. It was estimated that the IgA, IgG and IgM levels in saliva specimens were approximately 1/10, 1/800 and 1/400 of those measured in serum [55]. In urine, the detection of macromolecules such as IgGs (150 kDa), IgAs (320 kDA) and NS1 protein (300 kDa) most likely reflects an alteration of the glomerular filtration barrier which has three major components: the glomerular endothelial cells (GECs), the glomerular basement membrane and the podocytes. One of the characteristics of GECs is the presence of numerous fenestrations, which in theory should allow the passage of large molecules. GECs, however, are coated by a glycocalyx layer composed of glycosaminoglycans, which in normal conditions impedes the leakage of macromolecules. Abnormal disruption of the glycocalyx restores the passage of big molecules through the fenestration of the GECs [56]. High-molecular-weight proteins cannot pass through the podocytes barrier in normal conditions, but the accumulation of such macromolecules probably induces an alteration of this structure, which in turn leads to a proteinuria [57]. It has been suggested that during dengue infection, impairment of the glycocalyx layer on endothelial cells could occur. Both the virus itself and the NS1 antigen are known to fix heparan sulfate, a major glycosaminoglycan of the glycocalyx [58,59]. Moreover Wills *et al*. observed an increased urinary heparan sulfate excretion in children with severe dengue infection compared to healthy control subjects, suggesting that an alteration of the glycocalyx layer may occur in severe dengue infections [60]. The presence of circulating immune complexes formed to eliminate the virus and the NS1 antigen may provide another possible explanation to the alteration of the glomerular barrier. Cases of glomerulonephritis with glomerular immune complex-type deposits have been reported during DENV infections [61]. Jessie *et al*. also described the presence of viral antigen suggestive of the presence of immune complexes in the kidney tubular cells from dengue-infected patients [62]. Further investigations are needed on the potential interactions of dengue virus, immune complexes, and other components of the immune response with heparan sulfate and other glycosaminoglycan of the glycocalyx but also with the other kidney cells to determine if subclinical kidney lesions do not occur more often during DENV infection.

In this study we were not able to evaluate the performances of the different diagnostic methods for all four dengue virus serotypes as it was conducted using well-characterized and sequential clinical samples prospectively collected during a DENV-1 epidemic, when the DENV-2 and DENV-4 were circulating at lower level and no DENV-3 was detected.

The results of our study demonstrate that the diagnosis of dengue infection in urine and saliva specimens is possible but we believe that there is certainly still a room for improvement of these assays. Existing commercial rapid diagnostic tests (RDTs) designed to be used with blood samples would probably require significant adjustments, as urine and saliva specimens are very different from whole blood, plasma or serum. One of the main differences resides in the huge difference in protein concentration. A Singaporean group has recently developed a rapid test for the detection of anti-DENV IgG in saliva. The device gave good results in saliva samples spiked with IgG but requires further optimization to detect IgG from the clinical samples of dengue-infected patients [63]. Recently, saliva samples were tested using a commercial ELISA initially developed for IgG detection in blood and demonstrated 100% sensitivity and specificity [64].

In conclusion, although the performances of the different diagnostic methods evaluated here were not as good in saliva and urine as in plasma, results obtained with qRT-PCR and with antibody detection could justify the use of these two body fluids for the diagnosis of dengue infection for instances such as outbreak investigations or in young children (once they are old enough to comply to instructions), in addition to the situations when blood cannot be easily collected (e.g., lack of phlebotomist, refusal of the procedure, etc.). The disadvantage resulting from a slight decrease in the diagnostic confirmation performances when using molecular and serological tests in urine and saliva samples instead of blood is partially balanced by the ease to obtain these specimens and by the better compliance of the patient or by the number of individuals that can be investigated during studies when non-invasive sample collection methods are used. Moreover, the modest sensitivity of the test can be offset by high prevalence during the peak of an outbreak and/or when guided by competent clinicians. It results in high predictive positive value, making this test a useful addition for biological diagnosis in field conditions.

## Supporting Information

S1 Checklist STARD checklist. (DOCX)

S1 Fig Validation of the cut-off values of the saliva-based MAC-ELISA (A), AAC-ELISA (B), the urine-based AAC-ELISA (C), the saliva-based IgG indirect ELISA (D) and the urine- based IgG indirect ELISA (E). (PDF)

S2 Fig Mean NS1 concentration measured by capture ELISA in plasma, urine and saliva. (PDF)

S3 Fig Partial dependence plots for the most influential variables explaining NS1 concentration and RNA load in plasma. (PDF)

S1 Table Detailed protocols used in the NS1 capture ELISAs designed for plasma, urine and saliva specimens testing. (DOC)

S2 Table Protocols of the plasma-, urine- and saliva-based anti-DENV IgM capture ELISAs (MAC-ELISAs). (DOC)

S3 Table Protocols of the plasma-, urine- and saliva-based anti-DENV IgA capture ELISAs (AAC-ELISAs). (DOC)

S4 Table Intra-assay precision of the different ELISAs used in the study. (DOC)

S5 Table Inter-assay precision of the different ELISAs used in the study. (DOC)

S6 Table Protocols of the plasma-, urine- and saliva-based Indirect ELISAs for anti-DENV IgG detection. (DOC)

S7 Table Detection rate of viral genome, NS1 protein, anti-DENV antibodies in plasma, urine and saliva by time of sampling after onset of fever. (DOC)

S8 Table Results of the Boosted Regression Trees analysis for factors associated with NS1 concentration and viral RNA load. (DOC)

S9 Table Raw data. (XLSX)
