# Supplementary material for: Value of Routine Dengue Diagnostic Tests in Urine and Saliva Specimens
Source: PLoS Negl Trop Dis. 2015 Sep 25;9(9):e0004100. doi: 10.1371/journal.pntd.0004100 (PMC4583371; doi:10.1371/journal.pntd.0004100)

S1 Figure. Validation of the cut-off values of the saliva-based MAC-ELISA (A), AAC-ELISA (B), the urine-based AAC-ELISA (C), the saliva- based IgG indirect ELISA (D) and the urine- based IgG indirect ELISA (E). OD values for the different assays in saliva and urine specimens are divided into negative control and three other groups (low, medium, high) based on the titer of the corresponding antibody that was measured in the plasma of the patients. Cut-off values are indicated by the horizontal red line.

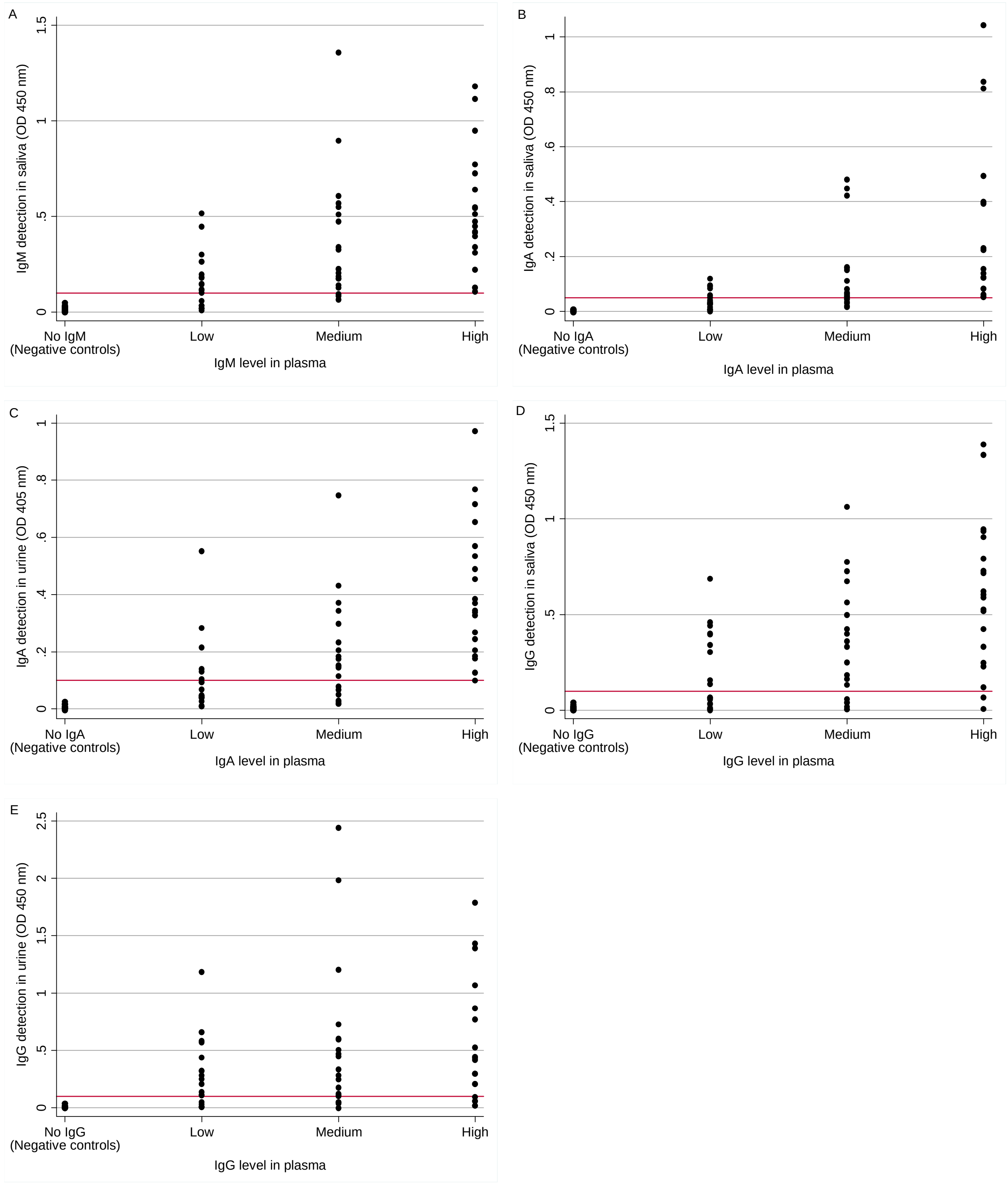

Supplement: S1 Fig — (PDF) [file pntd.0004100.s002.pdf]
