# Supplementary figures and images for: Value of Routine Dengue Diagnostic Tests in Urine and Saliva Specimens
Source: PLoS Negl Trop Dis. 2015 Sep 25;9(9):e0004100. doi: 10.1371/journal.pntd.0004100 (PMC4583371; doi:10.1371/journal.pntd.0004100)

S2 Figure. Mean NS1 concentration measured by capture ELISA in plasma, urine and saliva.

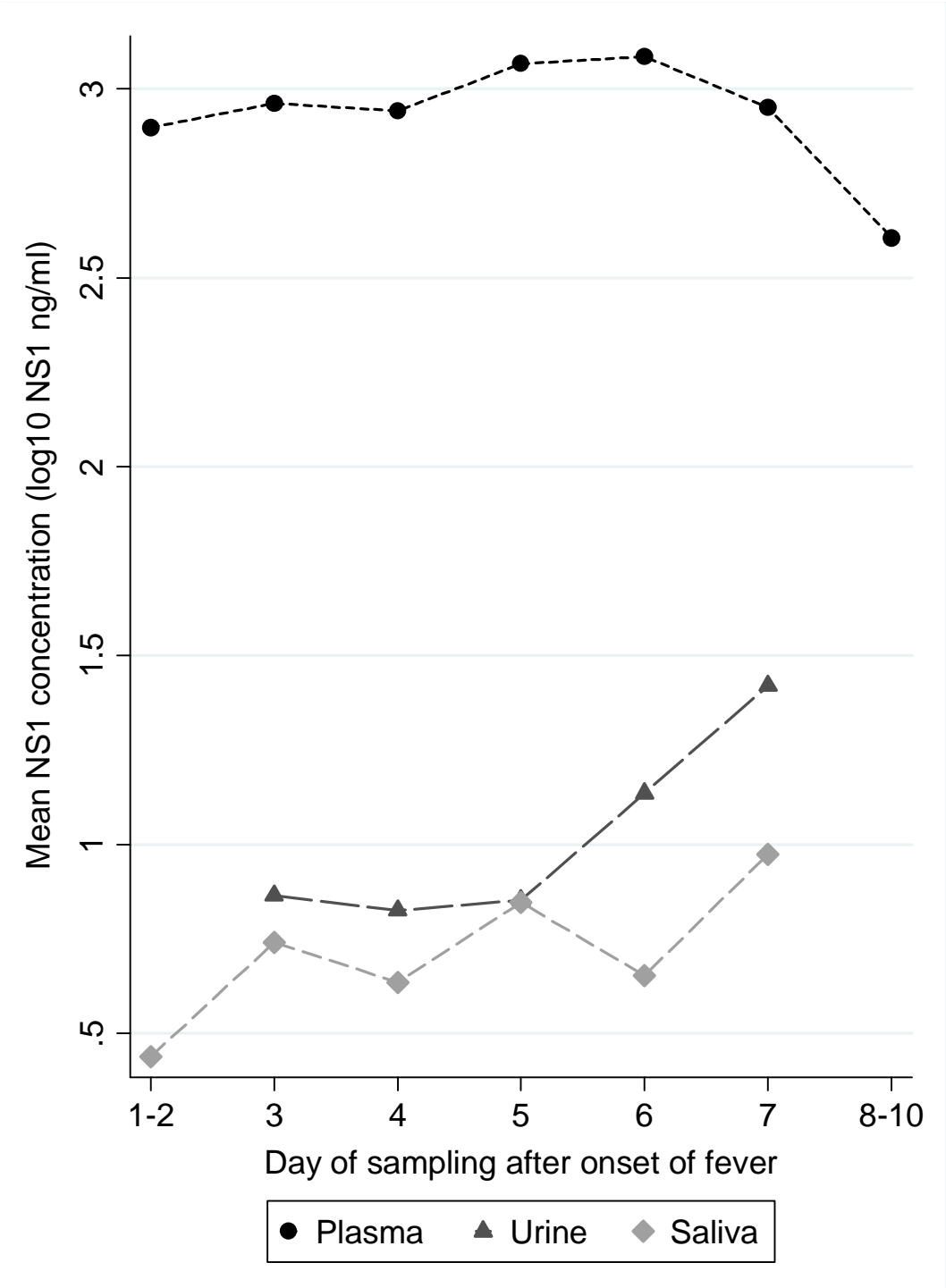

Supplement: S2 Fig — (PDF) [file pntd.0004100.s003.pdf]
