# Supplementary material for: Value of Routine Dengue Diagnostic Tests in Urine and Saliva Specimens
Source: PLoS Negl Trop Dis. 2015 Sep 25;9(9):e0004100. doi: 10.1371/journal.pntd.0004100 (PMC4583371; doi:10.1371/journal.pntd.0004100)

S3 Figure. Partial dependence plots for the most influential variables explaining NS1 concentration and RNA load in plasma.

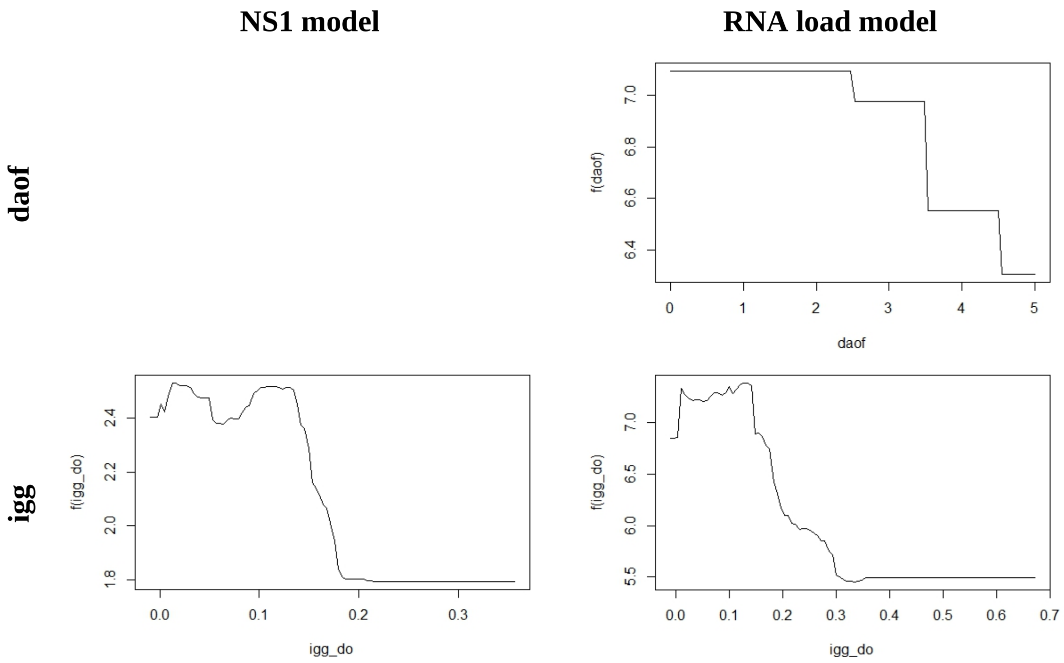

Supplement: S3 Fig — (PDF) [file pntd.0004100.s004.pdf]
