# Supplementary material for: Value of Routine Dengue Diagnostic Tests in Urine and Saliva Specimens
Source: PLoS Negl Trop Dis. 2015 Sep 25;9(9):e0004100. doi: 10.1371/journal.pntd.0004100 (PMC4583371; doi:10.1371/journal.pntd.0004100)
Supplement: S1 Table — (DOC) [file pntd.0004100.s005.doc]

**S1 Table. Detailed protocols used in the NS1 capture ELISAs designed for plasma, urine and saliva specimens testing.**

| Step | Details | Plasma | Urine | Saliva |
| --- | --- | --- | --- | --- |
| Coating | Reagent | In house monoclonal anti-NS1 antibody | In house monoclonal anti-NS1 antibody | In house monoclonal anti-NS1 antibody |
|  | Dilution | 2 µg/ml in PBS | 2 µg/ml in PBS | 2 µg/ml in PBS |
|  | Plate | Maxisorp (Nunc, Denmark) | Maxisorp (Nunc, Denmark) | Maxisorp (Nunc, Denmark) |
|  | Incubation (duration/temperature) | 4 hours/room temperature | 4 hours/room temperature | 4 hours/room temperature |
|  | Volume per well | 100 µl | 100 µl | 100 µl |
| Blocking | Reagent | Dilution buffer (PBS-M-T) | Dilution buffer (PBS-M-T) | Dilution buffer (PBS-M-T) |
|  | Incubation (duration/temperature) | 30 minutes/room temperature | 30 minutes/room temperature | 30 minutes/room temperature |
| Samples | Dilution | Non quantitative: 1/2 in PBS-M-T ; Quantitative : 1/2, 1/10 and 1/100 in PBS-M-T | 1/2 in PBS-M-T | 1/2 in PBS-M-T |
|  | Volume | 100 µl | 100 µl | 50 µl |
|  | Incubation (duration/temperature) | 1 hour/37°C | 1 hour/37°C | - * - |
| Conjugated antibody* | Reagent | Peroxidase-conjugated in house monoclonal anti-NS1 antibody | Peroxidase-conjugated in house monoclonal anti-NS1 antibody | Peroxidase-conjugated in house monoclonal anti-NS1 antibody |
|  | Dilution | 2 µg/ml in dilution buffer (PBS-M-T) | 2 µg/ml in dilution buffer (PBS-M-T) | 2 µg/ml in dilution buffer (PBS-M-T) |
|  | Volume | 100 µl | 100 µl | 50 µl |
|  | Incubation (duration/temperature) | 1 hour/37°C | 1 hour/37°C | 2 hour/37°C |
| Substrate | Reagent | TMB (KPL, USA) | TMB (KPL, USA) | TMB (KPL, USA) |
|  | Incubation | 10 min | 10 min | 10 min |
| Stop solution |  | H_2_SO_4_ 1N | H_2_SO_4_ 1N | H_2_SO_4_ 1N |

Dilution buffer (PBS-M-T): PBS containing 3% of skimmed milk and 0.1% of Tween 20

Between each step: 4 washes with a PBS solution containing 0.05% Tween 20

* For saliva testing, samples and conjugated antibody are distributed at the same step.
