# Supplementary material for: Value of Routine Dengue Diagnostic Tests in Urine and Saliva Specimens
Source: PLoS Negl Trop Dis. 2015 Sep 25;9(9):e0004100. doi: 10.1371/journal.pntd.0004100 (PMC4583371; doi:10.1371/journal.pntd.0004100)
Supplement: S2 Table — (DOC) [file pntd.0004100.s006.doc]

**S2 Table. Protocols of the plasma-, urine- and saliva-based anti-DENV IgM capture ELISAs (MAC-ELISAs).**

| Step | Details | Plasma* | Urine* | Saliva** |
| --- | --- | --- | --- | --- |
| Coating | Reagent | Goat anti-human IgM immunoglobulins (Sigma Aldrich, Germany) | Goat anti-human IgM immunoglobulins (Sigma Aldrich, Germany) | Goat anti-human IgM immunoglobulins (Sigma Aldrich, Germany) |
|  | Dilution | 1 µg/ml in PBS | 2 µg/ml in PBS | 2 µg/ml in PBS |
|  | Plate | Maxisorp (Nunc, Denmark) | Maxisorp (Nunc, Denmark) | Maxisorp (Nunc, Denmark) |
|  | Incubation (duration/temperature) | 4 hours/room temperature | 4 hours/room temperature | 4 hours/room temperature |
| Blocking | Reagent | Dilution buffer (PBS-M-T) | Dilution buffer (PBS-M-T) | Dilution buffer (PBS-M-T) |
|  | Incubation (duration/temperature) | 30 minutes/room temperature | 30 minutes/room temperature | 30 minutes/room temperature |
| Samples*** | Dilution | 1/100 in PBS-M-T | 1/5 in PBS-M-T | 1/5 in PBS-M-T |
| Antigen | Reagent | Positive antigen : sucrose-acetone extracted DENV-1, DENV-2 and DENV-3 antigens;  Negative antigen: brain of non infected mice | Positive antigen: sucrose-acetone extracted DENV-1, DENV-2 and DENV-3 antigens;  Negative antigen: brain of non infected mice | Positive antigen: sucrose-acetone extracted DENV-1, DENV-2 and DENV-3 antigens;  Negative antigen: brain of non infected mice |
|  | Dilution | 16 Hemagglutination units in dilution buffer (PBS-M-T) | 16 Hemagglutination units in dilution buffer (PBS-M-T) | 16 Hemagglutination units in dilution buffer (PBS-M-T) |
|  | Incubation (duration/temperature) | Overnight/4°C | Overnight/4°C | Overnight/4°C |
| Antibody 1 | Reagent | Mouse hyper-immune ascitis | Purified Mouse anti-Pan Dengue envelope protein, Clone D1-11 (ICL, USA) | Purified Mouse anti-Pan Dengue envelope protein, Clone D1-11 (ICL, USA) |
|  | Dilution | 1/1000 in dilution buffer (PBS-M-T) | 2 µg/ml in dilution buffer (PBS-M-T) | 2 µg/ml in dilution buffer (PBS-M-T) |
|  | Incubation (duration/temperature) | 1 hour/37°C | 1 hour/37°C | 1 hour/37°C |
| Antibody 2 | Reagent | Peroxidase-conjugated Fab anti-mouse IgG H+L (supplier) | Peroxidase-conjugated Fab anti-mouse IgG H+L (supplier) | Peroxidase-conjugated Fab anti-mouse IgG H+L (supplier) |
|  | Dilution | 1/5000 in dilution buffer (PBS-M-T) | 1/15000 in dilution buffer (PBS-M-T) | 1/15000 in dilution buffer (PBS-M-T) |
|  | Incubation (duration/temperature) | 1 hour/37°C | 1 hour/37°C | 1 hour/37°C |
| Substrate | Reagent | ABTS (KPL, USA) | TMB (KPL, USA)**** | TMB (KPL, USA)**** |
|  | Incubation | 10 min | 10 min | 10 min |

Dilution buffer (PBS-M-T): PBS containing 5% of skimmed milk and 0.5% of Tween 20

Between each step: 4 washes with a PBS solution containing 0.05% Tween 20

*Distribution of 100 µl/well at each step

** Distribution of 50 µl/well at each step

*** Each sample is tested twice: in one well with positive antigen and in one well with negative antigen

****Addition of H_2_SO_4_ 1N at the end of the incubation
