# Supplementary material for: Value of Routine Dengue Diagnostic Tests in Urine and Saliva Specimens
Source: PLoS Negl Trop Dis. 2015 Sep 25;9(9):e0004100. doi: 10.1371/journal.pntd.0004100 (PMC4583371; doi:10.1371/journal.pntd.0004100)
Supplement: S4 Table — (DOC) [file pntd.0004100.s008.doc]

**S4 Table. Intra-assay precision of the different ELISAs used in the study.** Intra-assay precision (repeatability) was assessed by testing one negative sample and three positive samples 10 times in the same assay.

|  | **Negative sample** | **Positive sample 1** | **Positive sample 2** | **Positive sample 3** |
| --- | --- | --- | --- | --- |
| **NS1 plasma** | | | | |
| Mean OD | 0.033 | 0.322 | 1.224 | 2.539 |
| SD | 0.001 | 0.017 | 0.031 | 0.045 |
| **NS1 urine** | | | | |
| Mean OD | 0.030 | 0.220 | 1.074 | 2.523 |
| SD | 0.003 | 0.008 | 0.024 | 0.043 |
| **NS1 saliva** | | | | |
| Mean OD | 0.024 | 0.130 | 0.495 | 1.260 |
| SD | 0.001 | 0.014 | 0.015 | 0.081 |
| **MAC-ELISA plasma** | | | | |
| Mean OD | 0.009 | 0.299 | 0.816 | 0.956 |
| SD | 0.002 | 0.009 | 0.028 | 0.015 |
| **MAC-ELISA saliva** | | | | |
| Mean OD | 0.015 | 0.099 | 0.698 | 1.063 |
| SD | 0.002 | 0.003 | 0.020 | 0.014 |
| **AAC-ELISA plasma** | | | | |
| Mean OD | 0.001 | 0.450 | 0.631 | 1.094 |
| SD | 0.002 | 0.007 | 0.016 | 0.015 |
| **AAC-ELISA urine** | | | | |
| Mean OD | 0.005 | 0.354 | 0.558 | 0.714 |
| SD | 0.003 | 0.023 | 0.036 | 0.020 |
| **AAC-ELISA saliva** | | | | |
| Mean OD | 0.004 | 0.110 | 0.952 | 1.420 |
| SD | 0.003 | 0.004 | 0.018 | 0.018 |
| **IgG indirect ELISA plasma** | | | | |
| Mean OD | 0.002 | 0.249 | 0.517 | 0.574 |
| SD | 0.002 | 0.017 | 0.016 | 0.015 |
| **IgG indirect ELISA urine** | | | | |
| Mean OD | 0.015 | 0.194 | 0.511 | 1.826 |
| SD | 0.001 | 0.004 | 0.009 | 0.035 |
| **IgG indirect ELISA saliva** | | | | |
| Mean OD | 0.014 | 0.141 | 0.222 | 0.481 |
| SD | 0.002 | 0.004 | 0.009 | 0.022 |

SD: Standard Deviation
