# Supplementary material for: Value of Routine Dengue Diagnostic Tests in Urine and Saliva Specimens
Source: PLoS Negl Trop Dis. 2015 Sep 25;9(9):e0004100. doi: 10.1371/journal.pntd.0004100 (PMC4583371; doi:10.1371/journal.pntd.0004100)
Supplement: S5 Table — (DOC) [file pntd.0004100.s009.doc]

**S5 Table. Inter-assay precision of the different ELISAs used in the study. Inter-assay precision (reproducibility) was assessed by testing one negative sample and three positive samples 5 times in different assays.**

|  | **Negative sample** | **Positive sample 1** | **Positive sample 2** | **Positive sample 3** |
| --- | --- | --- | --- | --- |
| **NS1 plasma** | | | | |
| Mean OD | 0.066 | 0.364 | 1.211 | 2.476 |
| SD | 0.020 | 0.027 | 0.043 | 0.051 |
| **NS1 urine** | | | | |
| Mean OD | 0.090 | 0.240 | 1.082 | 2.486 |
| SD | 0.037 | 0.006 | 0.042 | 0.088 |
| **NS1 saliva** | | | | |
| Mean OD | 0.034 | 0.132 | 0.488 | 1.255 |
| SD | 0.009 | 0.010 | 0.017 | 0.077 |
| **MAC-ELISA plasma** | | | | |
| Mean OD | 0.008 | 0.247 | 0.780 | 0.913 |
| SD | 0.003 | 0.044 | 0.120 | 0.123 |
| **MAC-ELISA saliva** | | | | |
| Mean OD | 0.017 | 0.103 | 0.677 | 1.091 |
| SD | 0.004 | 0.005 | 0.054 | 0.113 |
| **AAC-ELISA plasma** | | | | |
| Mean OD | 0.002 | 0.465 | 0.665 | 1.151 |
| SD | 0.001 | 0.039 | 0.056 | 0.117 |
| **AAC-ELISA urine** | | | | |
| Mean OD | 0.003 | 0.372 | 0.571 | 0.818 |
| SD | 0.002 | 0.068 | 0.067 | 0.087 |
| **AAC-ELISA saliva** | | | | |
| Mean OD | 0.004 | 0.093 | 0.905 | 1.330 |
| SD | 0.004 | 0.011 | 0.063 | 0.078 |
| **IgG indirect ELISA plasma** | | | | |
| Mean OD | 0.009 | 0.267 | 0.472 | 0.588 |
| SD | 0.015 | 0.042 | 0.050 | 0.089 |
| **IgG indirect ELISA urine** | | | | |
| Mean OD | 0.016 | 0.167 | 0.433 | 1.517 |
| SD | 0.004 | 0.015 | 0.044 | 0.162 |
| **IgG indirect ELISA saliva** | | | | |
| Mean OD | 0.012 | 0.150 | 0.229 | 0.497 |
| SD | 0.002 | 0.020 | 0.020 | 0.049 |

SD: Standard Deviation
