# Supplementary material for: Value of Routine Dengue Diagnostic Tests in Urine and Saliva Specimens
Source: PLoS Negl Trop Dis. 2015 Sep 25;9(9):e0004100. doi: 10.1371/journal.pntd.0004100 (PMC4583371; doi:10.1371/journal.pntd.0004100)
Supplement: S7 Table — (DOC) [file pntd.0004100.s011.doc]

**S7** Table. Detection rate of viral genome, NS1 protein, anti-DENV antibodies in plasma, urine and saliva by time of sampling after onset of fever.

|  | **PCR plasma** | **PCR urine** | **PCR saliva** | **NS1 plasma** | **NS1 urine** | **NS1 saliva** | **IgM/IgG/IgA plasma** | **IgG/IgA urine** | **IgM/IgG/IgA saliva** |
| --- | --- | --- | --- | --- | --- | --- | --- | --- | --- |
| **Total** | 85.4% (323/378) | 41.6% (184/442) | 39.0% (219/562) | 63.4% (338/533) | 14.5% (124/856) | 28.3% (195/688) | 36% (244/678); 54.4% (417/766); 37.4% (291/778) | 38.5% (575/1493); 26.8% (397/1483) | 38.1% (531/1395); 52.9% (594/1123); 28.6% (315/1101) |
| **D≤2** | 100% (37/37) | 13.6% (3/22) | 60.5% (23/38) | 82.4% (56/68) | 3.2% (2/63) | 15.4% (8/52) | 1.7% (1/59); 14.7% (10/68); 0% (0/70) | 3.9% (3/78); 0% (0/78) | 1.6% (1/63); 8% (4/50); 0% (0/48) |
| **D3** | 100% (54/54) | 23.8% (10/42) | 63.9% (39/61) | 88.2% (75/85) | 15.1% (14/93) | 33.3% (27/81) | 7.6% (6/79); 15.9% (13/82); 6.9% (6/87) | 7.3% (8/110); 0.9% (1/110) | 8% (8/100); 12.7% (9/71); 1.4% (1/72) |
| **D4** | 96.0% (72/75) | 41.1% (30/73) | 58.5% (55/94) | 75.5% (80/106) | 24.3% (33/136) | 42.6% (49/115) | 22% (20/91); 36.5% (39/107); 18.2% (20/110) | 20.3% (33/163); 8.6% (14/163) | 20.4% (29/142); 20.9% (24/115); 7.6% (8/106) |
| **D5** | 91.2% (52/57) | 48.2% (40/83) | 44.2% (46/104) | 62.3% (48/77) | 23.5% (34/145) | 31.1% (41/132) | 43.8% (28/64); 47.2% (34/72); 42.3% (33/78) | 33.2% (61/184); 21.2% (39/184) | 42.1% (72/171); 35.4% (46/136); 23.3% (31/133) |
| **D6** | 75.0% (36/48) | 50% (38/76) | 30.9% (30/97) | 51.7% (30/58) | 15.0% (20/133) | 27.3% (30/110) | 65% (39/60); 59% (46/78); 56.3% (45/80) | 48.9% (87/178); 35.4% (63/178) | 54.6% (83/152); 50% (63/126); 51.2% (65/127) |
| **D7** | 72.9% (35/48) | 43.9% (25/57) | 23.9% (17/71) | 49.1% (30/61) | 12.8% (14/109) | 26.1% (23/88) | 85.7% (54/63); 65.2% (45/69); 72.7% (51/70) | 58.7% (78/133); 46.2% (61/132) | 73.3% (96/131); 65.4% (68/104); 53.3% (57/107) |
| **W2** | 62.7% (37/59) | 49.3% (37/75) | 10% (9/90) | 24.4% (19/78) | 4.1% (7/172) | 15.4% (17/110) | 82.2% (83/101); 82.3% (93/113); 88.7% (102/115) | 70.2% (153/218); 64.2% (140/218) | 72% (144/200); 78.9% (131/166); 65.1% (110/169) |
| **W3** | NA | 7.1% (1/14) | 0% (0/3) | NA | 0% (0/5) | NA | NA | 56.2% (50/89); 51.7% (46/89) | 55.1% (48/87); 79.1% (53/67); 30.9% (21/68) |
| **W4** | NA | NA | 0% (0/4) | NA | NA | NA | NA | 46.5% (47/101); 23% (23/100) | 28.4% (25/88); 86.6% (58/67); 14% (10/71) |
| **W5** | NA | NA | NA | NA | NA | NA | NA | 37.9% (22/58); 10.5% (6/57) | 25.8% (17/66); 78.7% (37/47); 18% (9/50) |
| **W6** | NA | NA | NA | NA | NA | NA | 16% (12/75); 81.3% (65/82); 32.1% (25/78) | 25.9% (22/85); 3.6% (3/84) | 6.3% (6/95); 67.1% (57/85); 3.7% (3/82) |
| **M3** | NA | NA | NA | NA | NA | NA | 1.2% (1/86); 74.2% (72/97); 10% (9/90) | 11.5% (11/96); 1.1% (1/90) | 2% (2/100); 46.3% (44/95); 0% (0/68) |

D: day after the onset of the fever; W: week after the onset of the fever; M: month after the onset of the fever

NA: No sample Available
