# Supplementary material for: Value of Routine Dengue Diagnostic Tests in Urine and Saliva Specimens
Source: PLoS Negl Trop Dis. 2015 Sep 25;9(9):e0004100. doi: 10.1371/journal.pntd.0004100 (PMC4583371; doi:10.1371/journal.pntd.0004100)
Supplement: S8 Table — (DOC) [file pntd.0004100.s012.doc]

**S8 Table. Results of the Boosted Regression Trees analysis for factors associated with NS1 concentration and viral RNA load.**

|  | **Mean Relative Importance (Standard deviation)** | | | **Mean correlation coefficient (Standard deviation)** |
| --- | --- | --- | --- | --- |
|  | **daof** | **classif** | **IgG** |
| NS1 model (logns1) | 8.51 (1.03) | 6.30 (0.54) | 85.19 (1.49) | 0.505 (0.014) |
| Viral RNA model (logvir) | 20.87 (0.31) | 1.81 (0.19) | 77.32 (0.37) | 0.618 (0.002) |

daof: number of days between the onset of the fever and the time of sampling

classif: classification of the severity of the disease according to 1997 WHO guidelines

IgG: anti-DENV IgG quantity in plasma estimated by optical density in indirect ELISA
